# Factors Influencing Participation in Physical Activity, Sports, and Exercise in Children and Adolescents with Spinal Pain or Spinal Conditions: A Systematic Review and Meta-Ethnography

**DOI:** 10.3390/bs13060486

**Published:** 2023-06-09

**Authors:** Susanna Tucker, Nicola R. Heneghan, Adrian Gardner, Alison Rushton, Samia Alamrani, Andrew Soundy

**Affiliations:** 1 School of Sport, Exercise and Rehabilitation Sciences, University of Birmingham, Birmingham B15 2TT, UK; 2 Spinal Surgery, The Royal Orthopaedic Hospital NHS Foundation Trust, Birmingham B31 2AP, UK; 3 School of Physical Therapy, Western University Canada, London, ON N6A 3K7, Canada; 4 Physical Therapy Department, University of Tabuk, Tabuk 47512, Saudi Arabia

**Keywords:** adolescent, paediatric, thoracic, lumbar, spinal, pain, sports, exercise, physical, participation

## Abstract

Background: Physical activity is an effective treatment for paediatric spinal pain. However, participation rates remain low and review evidence is needed to establish why. This review identifies factors influencing participation in sports, exercise, and physical activity in those aged 18 or under with spinal pain or spinal conditions. Trends or differences between discrete sub-populations are identified. Methods: A meta-ethnographic review was undertaken. Qualitative papers were identified and appraised using the JBI checklist. Thematic trends were mapped onto the biopsychosocial model and subthemes identified. Uniqueness was calculated and the confidence in the evidence was evaluated using the GRADE-CERQual tool. Results: Data were gathered from nine qualitative papers (384 participants). Three themes were identified: (1) biological: physical challenges and bladder and bowel care; (2) psychological: perceptions of differences to peers, struggle, anger, sadness, adjustment, and acceptance; and (3) sociological: influence of friends, social acceptance, negative attitudes from others, and the influence of their disability on family routine. Conclusions: Sociological factors were most influential on exercise participation alongside related psychological and biological factors. Adolescents over 14 years offered greater critical insight compared to the younger children. Results are best applied to neuromuscular conditions with further robust evidence required in paediatric musculoskeletal spinal pain.

## 1. Introduction

Spinal pain is one of the leading causes of disability and presents significant cost to society through medical burden and absenteeism from work [1]. Whilst considerable attention has been given to the burden of spinal pain in the general population, there is an increasing interest in discrete populations, such as older adults and adolescents [2,3]. Recent evidence suggests that spinal pain often starts in childhood, affecting approximately 20% of adolescents, and continues to increase in prevalence and burden through adolescence into adulthood [2,4,5]. Thoracic spinal pain is estimated at around 35% in children aged 12, with 10% of adolescents stating that they experience thoracic pain that interferes with school or leisure [6]. Adolescent spinal pain has a significant influence on lifestyle, with up to 38% reporting an influence on participation in physical activity (PA), care-seeking, or activities of daily living [6]. Adolescent idiopathic scoliosis (AIS) is the most common adolescent spinal condition, present in approximately 2–3% of children under 16 years, and characterised by a three-dimensional deformity of the spine [7].

Due to the large volume of research in paediatric cervical pain and the existence of current guidelines, this systematic review and meta-ethnography has been limited to the gap in the literature surrounding paediatric thoracic and lumbar spinal pain [8,9,10]. This was primarily due to the varied and complex presentations of neck pain and its increasing prevalence with age [8]. Furthermore, paediatric cervical injury is one of the most common complaints, but with a unique and specific set of guidelines for assessment and treatment [9]. Due to the high prevalence of paediatric cervical neck pain and its associations with developmental, medical, psychological, and social complications [10], it was determined that these patients would not be included in this review. This was done in an attempt to further distinguish uniqueness in a widely under-researched group, namely, children and adolescents with thoracic and lumbar spinal conditions or pain.

The UK government and National Health Service (NHS) state that children and adolescents should engage in 60 min of PA per day, varying in type and intensity [11]. Behavioural treatments, such as exercise for spinal pain in children and adolescents, have been shown to increase quality of life, physical functioning, and reduce pain [12,13,14]. There is a clear trend between activity levels and back pain with studies showing that elite adolescent athletes who regularly participate in sports have a lower incidence of back pain compared to adolescent non-athletes or the general population [15]. The literature encourages PA in those with AIS regardless of whether there has been surgical input or not [16]. Furthermore, the literature makes clear that clinicians should be advising patients that no harm is associated with PA and scoliosis is not a reason to avoid exercise [16]. However, to the best of the authors’ knowledge, uptake of exercise for those with AIS remains low with a lack of qualitative data exploring the factors influencing participation, despite the known benefits of sports, exercise, and PA and its central role in care pathways [17,18,19,20,21,22,23]. In order to understand these factors there is a body of qualitative research [24,25,26,27,28,29,30,31,32]. Synthesis of this is important and can be easily structured around related frameworks, such as the biopsychosocial model [33] and the international classification of functioning disability and health, children, and youth (ICF-CY) [34].

The ICF-CY is accepted as a framework produced by the World Health Organisation (WHO) to measure health and disability [34]. Using the ICF-CY will provide an understandable assessment of functional complexity of the spinal condition in a holistic way using common language [35], aiding interpretation. Understanding of physical functioning, sports, and exercise can vary greatly amongst patients and professionals, therefore, it was determined necessary to define PA, sports, and exercise for the purposes of this study. The ICF-CY incorporates a biopsychosocial approach whilst being validated for use across multiple countries and cultures [36,37,38]. The ICF-CY is valuable at key transition points in a child’s life, identifying components of functioning that contribute to quality of life, thereby making it critical to this study [39].

The biopsychosocial model is one of the most widely accepted tools used to manage back pain due to its effectiveness in managing these complex patients with a holistic and multifaceted approach [33]. Although there is debate regarding the effectiveness of the biopsychosocial model due to the complex interactions of each component, it offers a more sophisticated perspective on care than the biomedical model and is widely used in the management of back pain [40,41]. Through mapping the results of this review onto the biopsychosocial model we can further understand each of the three components whilst allowing for commonality in factors influencing exercise, and thereby apply this to physiotherapeutic practice as opposed to creating a new model or process with results [42]. The benefits of PA, sports, and exercise and the unique, complex, and varied presentations of adolescent spinal pain requires therapists to take a holistic approach and ensure that psychological and sociological factors are addressed alongside biological factors [19,33,43]. However, many physiotherapists feel ill-equipped to manage these complex spinal pain patients and implementing the biopsychosocial model for exercise habituation [44,45,46].

Despite the prevalence of AIS, there is a substantial lack of primary evidence regarding qualitative factors influencing exercise. Therefore, this project aims to review the existing qualitative literature looking at factors influencing sports, exercise, and PA participation in children and adolescents aged 18 years or under with thoracic or lumbar spinal pain or diagnosed spinal conditions. A meta-ethnographic review is well positioned to bring together the complex experiences of individuals. It has been highlighted as useful for theory development [47] and for clinical guideline development [48].

### Objectives

(a) To describe factors influencing participation in sports, exercise, and PA in children and adolescents aged 18 years or under with thoracic or lumbar spinal pain or diagnosed conditions;
(b) To identify any trends or differences in factors influencing participation between discrete sub-populations, such as age, gender, or spinal condition.

## 2. Materials and Methods

**Figure 1:**
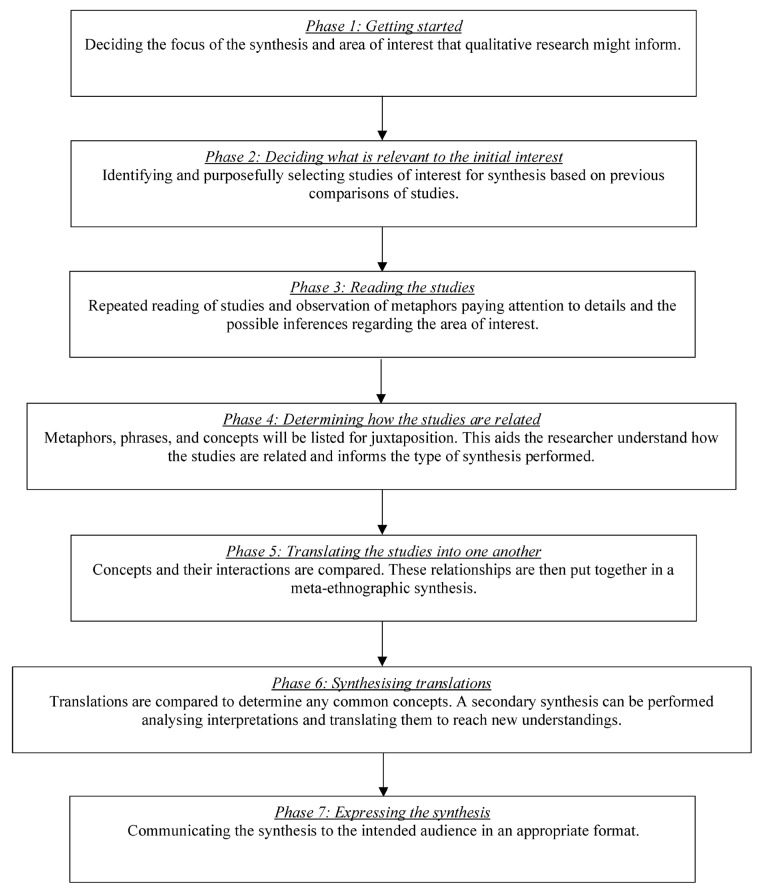
Illustrating the seven phases of meta-ethnography.

A meta-ethnographic review was designed according to the traditional seven stages of meta-ethnography (see Figure 1) [49] and the meta-ethnography reporting guidelines (eMERGe) [50]. This review was situated within a subtle realist world view [51] and designed to establish naturalistic generalisation [52], and inform clinical and research recommendations [53]. This review was registered with the international prospective register of systematic reviews (PROSPERO) (reference: CRD42022314796) [54].

### 2.1. Patient and Public Involvement

This research was conceived following a focus-group discussion with expert clinicians, academics, and a lead spinal surgeon working in the field of interest. The team supporting this review have expertise in spinal pain (NH, AR, and AG) and qualitative research (AS, AR, and NH), with all having expertise in conducting reviews for impact.

### 2.2. Eligibility Criteria

Phase one of Noblit and Hares meta-ethnography involves identifying the area of intellectual interest [55]. The search concept tool SPIDER (sample, phenomenon of interest, design, evaluation, and research type) informed study eligibility (Table 1) [56].

### 2.3. Information Sources

Ten electronic databases from inception until April 2022 were searched as follows: AMED, EMBASE, MEDLINE, CINAHL Plus, Sport Discuss, SCOPUS, PubMed, PsychInfo, Nursing & Allied Health Database, and Social Science Database.

Grey literature using reference lists, websites, and search engines, for example, Google scholar, was performed on 28 April 2022. This searching was undertaken in accordance with the Peer Review of Electronic Search Strategies guidelines [57].

### 2.4. Search Strategy

Outlined below in Table 2 is the search strategy, informed by previous scoping searches. Boolean operators such as ‘AND’ and ‘OR’ were utilised with Truncation (*) tailored for each database [56].

### 2.5. Data Management

All records were stored in EndNote20 to produce an audit trail [58]. Following this process, full texts were assessed for eligibility and to determine which will be included in the final review.

A PRISMA (preferred reporting items for systematic reviews and meta-analyses) diagram was used to outline the results produced at different stages of the literature search (Appendix A) [59]. Studies that contain qualitative data, e.g., barriers and facilitators of exercise in those with spinal pain, were identified for quality analysis [60].

### 2.6. Selection Process

Phase two of Noblit and Hare’s meta-ethnograhy is determining what is relevant to initial interest [55]. Study selection was determined by the first reviewer (ST) with consultation of the second reviewer at each stage (AS), and any disagreements discussed with the third reviewer (SA). Firstly, studies were screened by title, followed by the abstract, followed by full text, and selected against the predefined criteria.

During phase three, each of the studies’ participant quotes were read and analysed, and comments made using an open coding technique leaving an appropriate audit trail (Supplementary File S3) [47]. This process was performed blinded by the first researcher (ST) and the second researcher (AS). Studies and accounts were read with attention to phrases and metaphors, to determine if the study was relevant to phenomena of interest [55]. From the studies read, it was clear whether or not the study met the inclusion criteria and author contact was required or not. Phase three was completed by the first researcher (ST), alongside consultation with the second reviewer (AS). Any disagreements were resolved with involvement of a third reviewer (SA) [61].

### 2.7. Data-Collection Process

During phase four, studies were examined and the relationship between the studies determined for synthesis [55]. Key phrases, metaphors, ideas, and concepts were listed for juxtaposition [55]. Consideration of the studies’ theoretical approaches, concepts, themes, and metaphors informed the relationship of the studies and, therefore, the data synthesis [49,55]. A table was used to display commonalities with reciprocal translation and differences using refutational translation amongst concepts within and between studies [55].

This process was undertaken by the lead researcher (ST), with data checked by the second reviewer (AS), and disagreements discussed with the third reviewer (SA) [56]. Where there was insufficient or missing data the lead researcher made two attempts to contact the author via email over a four-week period.

### 2.8. Data Items

Phase five involved translating the studies into one another [55]. Comments or considerations from both the first (ST) and second researcher (AS) were compared, and consistent coding themes were generated for input into the next stage of the data synthesis. Due to the nature of the studies being about similar matters, they were able to reciprocally be added together [62].

This synthesis compared and identified the range of metaphors, concepts, or themes and their relationship across study accounts [55]. Translation was idiomatic rather than literal, taking account of each studies’ context, a central practice in meta-ethnography [49,55]. Reciprocal translation was used for studies containing similar themes allowing them to be ‘added together’ [49,63].

Results of individual studies are presented and tabulated below. Firstly, summary data for each study and the uniqueness of each group between studies are presented. Secondly, frequency of qualitative statements and proportions of different demographic groups are represented using statistical proportions to enable identification of generalisability and applicability. Data were checked by the second researcher (AS) for accuracy [55].

### 2.9. Risk of Bias in Individual Studies

Studies were critically appraised using the Joanna Briggs Institute (JBI) checklist for qualitative research critical appraisal tool (JBICQR), due to its associated validity and coherence [64,65]. Studies were selected based on their ability to answer the research question followed by quality appraisal, rather than initial exclusion based on quality [66]. This process was undertaken by the lead researcher (ST), with data checked by the second reviewer (AS) to establish consistency, with any concerns discussed with the third researchers (SA).

The trustworthiness of the included studies was critically appraised by the first researcher (ST) and discussed with the second researcher (AS). The JBICQR was used throughout data analysis for quality assessment (Supplementary File S1) [64,67]. Results from the JBICQR performed on each paper [64] were used to inform the certainty assessment performed using the confidence in the evidence from the reviews of qualitative research (GRADE-CERQual) tool [68]. This is further outlined and documented below in the results of the synthesis at the level of each subtheme. Quality has been presented according to between-study assessment and within-tool assessment. Each study included in the review has been cited and tabulated with the characteristics detailed.

### 2.10. Outcomes and Prioritisation

Each theme identified during phase five study translation had a determined effect measure with statistical proportions stated. For example, the number or participants who have the same response theme or the number of different responses identified.

Information on study characteristics, sample demographics, and qualitative responses were examined with conclusions extracted [47]. First-order constructs were determined related to participants quotations, and second-order constructs related to the researchers’ impressions [55]. The first researcher (ST) selected participant quotes to support second-order constructs, however, the difference between participant quotes and researcher impressions cannot be examined in isolation, therefore, allowing for interpretations to be made [55]. JBICQR and GRADE-CERQUAL appraisal tools used during the synthesis are included in the review appendices [56].

### 2.11. Synthesis Methods

During phase six, translations identified in stage five were compared to identify common overarching themes and subthemes, and develop interpretations [55]. Synthesis results are visually displayed below and tabulated (Supplementary File S3) with between-study and characteristics and uniqueness in demographic groups identified for themes and subthemes [55]. This phase enabled the first researcher (ST) to determine final themes alongside discussion with the second (AS) and third researchers (SA) to help reduce bias and errors in data extraction [55,56]. Due to the nature of meta-ethnography, multiple lines of argument were identified and combined into one theory, namely, the biopsychosocial model [55]. It was determined that qualitative factors influencing exercise are best understood alongside the biopsychosocial model of care as this model is used to underpin clinical practice, therefore, qualitative factors were mapped onto this model [19,33]. Thematic synthesis has enabled conclusions to be drawn based on common elements across heterogeneous studies [56]. This section includes details on the type of data extracted including population characteristics due to their influence on generalisability [56]. During this stage, studies were re-read to ensure interpretations were grounded within original studies.

Finally, phase seven involved expressing the synthesis by translating findings into a suitable format for the audience [63]. Outlined below are each of the three themes from the biopsychosocial model, followed by a definition of the subtheme, example quotes, and a brief explanation of uniqueness. According to the biopsychosocial model framework, three titles were used: biological factors (focusing on the mechanical and physiological impact of biological factors), psychological (focusing on thoughts, feelings, and beliefs in relation to subjective experience), and social (focusing on the impact of condition on socialising, family, and friendships, and access to group activities) [40]. Subthemes were identified within each section. Uniqueness for each subtheme was presented according to age, gender, condition, or differences between parent and child quotations. In the results below, children are described as 0–13 years and adolescents 14–18 years.

For this review it was necessary to divide participants into demographic groups dependent on their age in order to identify themes and uniqueness. The two groups identified were children aged 0–13 years and adolescents aged 14–18 years. The decision to separate the children from the adolescents at 14 years was based on the care-quality commission (CQC) guidelines stating that the transition to adult services should begin at 14 years [69]. For those in the United Kingdom (UK), transition to adult services must occur prior to the cut-off point between 16 and 18 years, case-dependent [70]. Although this review identified a number of the papers from a variety of countries around the world, it appears that only the UK and Australia have national guidance on age of transition, with other countries relying on academics in speciality groups to provide guidance [71]. It is clear that there is debate amongst professionals and inconclusive literature regarding the specific age of transition from childhood to adolescence but a general consensus is around puberty [72]. The World Health Organisation (WHO) states that adolescence is between the ages of 10 and 19 years of age, with key social transitions taking place after biological maturity and menarche [73,74]. The United Nations International Treaty states that an individual remains classified as a child whilst under the age of 18 years [75].

Further dissemination will be done through publication and conference presentations. Furthermore, the meta-synthesis produced can be utilised for the underpinning and justification of health policy, therefore, expression for stakeholders is of the upmost importance in moving the physiotherapy profession forwards [76].

### 2.12. Reporting Bias and Trustworthiness Assessment

Quality of data, robustness, and risk of bias was assessed first for each paper using the JBICQR [64]. See a copy of the JBI checklist for each of the included studies in Supplementary File S1. Risk of bias was then assessed for each subtheme using the GRADE-CERQual certainty assessment [68] (Supplementary File S4). Methodological and analytical transparency were promoted throughout to enable the reader to determine if reasonable arguments and judgements are made [77]. All results are in Supplementary Files S1 and S2, and it is clarified to the reader that this is to help reduce reporting bias [78].

### 2.13. Certainty Assessment

Assessment of reporting bias from missing results in the synthesis has been clarified throughout the stages of the meta-ethnography. All results from the synthesis have been tabulated with a summary of meta-ethnography performed. Certainty assessment using GRADE-CERQUAL is stated at the level of the subtheme to enable differentiation in certainty of evidence and clarification for the reader [68]. See further details in Supplementary Files S2 and S4.

GRADE-CERQUAL was used to assess the quality of review findings by the theme generated from the synthesis. The assessment considers rating the level of concern (no concern or very low, low, moderate, and serious) across four criteria: (a) methodological limitations (using JBI quality assessment), (b) relevance, (c) accuracy, and (d) coherence. These four elements are brought together to determine confidence in evidence across a four-point scale (high, moderate, low, or very low confidence) [68].

## 3. Results

### 3.1. Study Selection

From the 223 articles screened, 9 were included. Table 3 contains participant details, please see Appendix A for the PRISMA flow diagram [59].

### 3.2. Study Characteristics

Table 3 shows details of each of the included studies and their respective characteristics. From the nine studies included in the final review, six examined participants with spina bifida, one examined spinal-cord injury, one examined achondroplasia, and one examined adolescents following spinal fusion surgery for scoliosis. Eight of the nine studies contained quotes from participants under 18 years and four of the nine studies contained quotes from children or adolescents and their parents or caregivers. Each study had a mixture of male and female participants from a variety of locations around the world (Canada, USA, Spain, The Netherlands, Palestine, South Africa, and Sweden).

Each included study contained qualitative data regarding uniqueness of children or adolescents with a diagnosed spinal condition or spinal pain and their experience of active participation in sports, exercise, PA, or functioning. Two studies contained information on demographics, such as household income, two studies contained information on ambulatory status, one study contained information on language of participants, and two studies contained information on participants’ beliefs or religion.

**Table 3:** Study Characteristics.

Study	Participant Characteristics (n = Children, p = Parents, c = Caregivers)	Participant Gender (F n = Female Children, M n = Male Children, F p = Female Parents, M p = Male Parents, F c = Female Caregivers, M c = Male Caregivers)	Participant Age (Years)	Participant Diagnosis	Setting
Bloemen, et al. (2015) [24]	4–7 years olds: n = 11 p =13 8–18 years: c = 33 p = 31	4–7 years olds: F n = 4 M n = 7 8–18 years olds: F n = 15 M n = 18	n = 11 × 4 to 7 n = 33 × 8 to 18	Spina bifida	A rehabilitation centre, a paediatric physiotherapy institution, or in the home situation in The Netherlands.
Gorzkowski, et al. (2011) [25]	c = 201	F n = 52% M n = 48%	n mean age at interview = 12.60 n mean age at injury = 7.19	Spinal-cord injury	Three paediatric spinal- cord injury centres.
Nahal, et al. (2019) [26]	n = 10	F n = 4 M n = 6	n = 7 to 18	Spinal bifida	Participants were recruited from three main rehabilitation centres from the north (n = 3), the middle (n = 3), and the south (n = 4) of the West Bank in Palestine.
Page and Coetzee (2021) [27]	n = 7	F n = 2 M n = 5 F c = 2 M c = 5	n = 13–16 n mean age = 14.5 c = 39–75 c mean age = 49.5	Spinal bifida	The Cape Flats, southeast of central Cape Town, South Africa.
Pfeiffer, et al. (2021) [28].	n = 32	F n = 20 M n = 12	n = 9 to 17 n mean =13 n = 13 × 9 to 12 n = 9 × 12 to 15 n = 10 × 15 to 18	Achondro-plasia	16 participants from the USA, and 16 participants from Spain.
Volfson, et al. (2020) [29].	n = 9	F n = 6 M n = 3	n = 1 × 14 n = 2 × 15 n = 3 × 16 n = 3 × 17 n mean = 15.9	Spina bifida	Spina bifida outpatient clinic at a rehabilitation centre located in a large urban centre in Canada.
Willson, et al. (2021) [30].	n = 8	F n = 4 M n = 4	n = 14 to 17 n mean = 15.4	Returning to PA following spinal surgery	Unclear
Fischer, et al. (2015) [31].	n = 11 10 parents	F n = 7 M n = 4 F p = 7 M p = 3	n = 1 × 6 n = 1 × 8 n = 1 × 9 n = 3 × 10 n = 1 × 14 n = 1 × 15 n = 1 × 16 n = 1 × 17 n = 1 × 18	Spina bifida	Spina bifida clinic at a large paediatric rehabilitation hospital in Canada.
Strömfors, et al. (2017) [32].	n = 8	F n = 4 M n = 4	n = 1 × 10 n = 1 × 11 n = 1 × 12 n = 2 × 14 n = 1 × 15 n = 2 × 17	Spina bifida	Swedish children and adolescents. Recruitment took place at a bowel- and bladder-functions clinic for children and adolescents with SB. Two participants completed interviews at home. Six participants completed interviews at a local health-care facility that they visited regularly.

### 3.3. Study Trustworthiness

Despite some methodological limitations identified, there was a low risk of bias for each of the included studies as evaluated by the JBICQR [67]. Two studies deemed to be unclear were identified. The first by Gorzkowski et al. [25], and the second by Pfeiffer et al. [28]. In both studies, qualitative statements from participants are represented as percentage proportions of participants rather than direct quotes. Therefore, although quotes are included from both Gorzkowski et al. [25] and Pfeiffer et al. [28], in achieving qualitative data saturation for each subtheme, calculation of gender or age uniqueness proved challenging in subthemes that contained these two papers due to participants’ unknown gender or age. For full information on quality assessment, see Supplementary File S1 [67].

### 3.4. Results of Individual Studies

Results of individual studies have been synthesised and included in the synthesis. Please see Supplementary File S3 for further information.

### 3.5. Results of Synthesis

Three master themes and 13 subthemes were determined during the fourth stage of meta-ethnography, and used to present the results [49]. See Table 4 for a summary of the themes. For further information on themes identified, calculation of uniqueness, and all participant quotes, please see Supplementary Files S2 and S3.

The results of the synthesis have been certainty assessed using GRADE-CERQual [68]. This has been further outlined and documented below in the results of the synthesis at the level of each subtheme. 

#### 3.5.1. Theme 1: Biological Factors

This theme was identified according to Kamper, et al. [33] and defined as the mechanical and physiological impact of biological factors, such as genetics, developmental abnormalities, or injuries [79]. Any impairment in body function or structure as a significant deviation or loss [34]. Two subthemes were identified within this theme.

Subthemes: 

##### Subtheme 1: Challenges to PA, Sports, or Exercise Participation Resulting from Biological Aspects of Physical Condition

This subtheme is supported by four studies. All participants consistently expressed concerns about the physical differences between themselves and their asymptomatic peers regarding ambulation, mobility, back pain, and how it influences sports, exercise, and PA. All participants in this subtheme had been diagnosed with spina bifida.

Example: 

*‘If he wants to go to a sledge hockey camp overnight and this kind of thing for a week, that’s the kind of thing you need to have more independence with’*

[31].

*‘… but still being aware if there is back pain… how much of it’*

[29].

Uniqueness: 

Nine quotes were identified for this subtheme. This subtheme was found to be particularly unique to children (n = 3/9, 33%) or adolescents (n = 6/9, 67%). Children and adolescents appeared to have a high self-efficacy, a desire to be active, and overcome physical challenges, rather than perceived as different from their peers due to their limitations. One negative case was identified by a child whose focus was on pain restricting or limiting participation in PA rather than increasing independence in order to become physically active.

All parents (n = 3/9, 33%) appeared to have a degree of scepticism and pragmatism about their child’s ability to participate, and overcome the physical challenges of sports, exercise, and PA participation.

Compared to other age groups, children aged 10–13 years (n = 3/9, 33%) had a greater desire to participate with their peers in sports, exercise, and PA. Comparatively, the adolescents aged 14–18 years (n = 6/9, 67%) appeared to have greater awareness of their physical challenges of participation in sports, exercise, and PA. However, the adolescents still exhibited a desire to participate alongside their peers where possible.

CERQual Summary: 

The criteria rating for this subtheme was rated as follows: (a) methodological limitations—minor concerns, (b) coherence—minor concerns, (c) adequacy of data—no concerns, and (d) relevance—moderate concerns. Concerns arose due to some lack of clarity surrounding the influence of the researcher on the research and all participants have spina bifida causing moderate concerns regarding relevance to adolescent spinal pain (Supplementary File S4). Confidence statement: given the assessment above, there was moderate confidence in evidence from this subtheme. The evidence may be most applicable to children with spina bifida.

##### Subtheme 2: Bladder and Bowel Care and Its Impact on Participation in PA, Sports, and Exercise

This subtheme is supported by five studies. Bladder and bowel care was discussed as a large obstacle to participation in PA or exercise. Participants explained the stigma that they believed was attached to incontinence and how it decreased their desire and ability to participate in sports and exercise. Furthermore, the practicalities and timing of completing bladder and bowel care limited their opportunities to participate.

Example: 

*‘whenever I run [my bladder] just lets go’ (10 years, female)*

[31].

*‘The reason that I have not told my friends, is that I see how they are now. Young people, and now I mean people my age, they are kind of, what can I say, immature, or they say certain things. They think that if a person is different, then that person is a freak or something like that. That is why I have not dared [to tell them about having SB]’ (15 years, male)*

[32].

*‘Everything is difficult in my life. I feel tired of living with incontinence and diapers… I hate the catheterization… I wondered why children like me with spina bifida should stay alive’ (12 years, female)*

[26].

Uniqueness: 

From the 18 quotes identified on this theme, 17 related to spina bifida and one to spinal cord injury.

The younger children aged 8–9 years (n = 3/18, 17%) discussed striving for independence and completing catheterisation autonomously. Children aged 10–13 years (n = 3/18, 17%) expressed more negative emotion towards incontinence and a desire to be continent the same as their peers. Older children aged between 14–18 years (n = 10/18, 56%) expressed a desire for secrecy and worry about negative comments or ridicule from peers. Similar to the younger children, the 14–18-year-olds expressed a desire for independence with incontinence care to facilitate participation in school activities.

Comments from the parents (n = 3/18, 33%) consistently expressed a desire for their child to be achieve independence with bladder and bowel care and to join in activities with their friends. Direct quotes from children (n = 4/18, 22%) and adolescents (n = 8/18, 44%) emphasised their desire for secrecy surrounding their incontinence for fear of ridicule and feeling that everything is difficult.

One negative case of spinal cord injury (n = 1/18, 6%) was identified with a participant stating that disability-related obstacles included the bowel program. All other patients had been diagnosed with spina bifida.

CERQual Summary: 

The criteria rating for this subtheme was as follows: (a) methodological limitations—minor concerns, (b) coherence—minor concerns, (c) adequacy of data—no concerns, and (d) relevance—no concerns. Very minor concerns have been identified due to lack of representation of data in Gorzkowski et al. [25]. Other data appear well represented with numerous quotes and variety of populations used. The majority of studies in this subtheme relate to spina bifida and one relating to spinal-cord injury. Studies have good methodological quality with one study only including limited participant quotes and, thereby, limited representation (Supplementary File S4). Confidence statement: given the assessment above, there were very minor concerns regarding the evidence for this subtheme.

#### 3.5.2. Theme 2: Psychological Factors

This theme was identified according to Kamper et al. [33] and defined as influence of thoughts, feelings, or emotions on behaviour [79]. Four subthemes were identified within this theme.

Subthemes: 

##### Subtheme 3: Feelings of Struggle and Needing for Physical Assistance from Others; Desire for Independence When Participating in Sports, Exercise, and PA

This subtheme is supported by five studies. Participants highlighted their need for assistance from parents or caregivers and feelings surrounding physical activities. Parents and caregivers discussed their perception of their child’s physical limitations.

Example: 

*‘I would like to join a public sports club. I love swimming and playing football with other children… I am very sad… It is difficult to reach these areas… I am not allowed to participate… I prefer to stay home’ (16 years, male)*

[26].

Uniqueness: 

Nine quotes were identified for this subtheme. Male participants (n = 4/9, 44%) expressed an awareness of inability to do things and a focus on the daily struggle resulting from physical limitations. Female participants (n = 3/9, 33%) focused on striving for autonomy and a desire to move away from dependence on others towards independence.

Older children aged 14–18 years (n = 6/9, 66%) identified and focused on achieving the independence necessary for adult life, work, or university. No consistent themes were identified for other ages.

All children (n = 7/9, 77%) stated awareness of need for assistance with tasks, and a desire to be independent with reaching, sporting activities, or toileting.

CERQual Summary: 

The criteria rating for this subtheme was as follows: (a) methodological limitations —minor concerns, (b) coherence—minor concerns, (c) adequacy of data—no concerns, and (d) relevance—no concerns. Four studies looking at spina bifida and one at achondroplasia. Minor concerns with methodological quality due to an unclear location of the researcher culturally and theoretically (Supplementary File S4). Confidence statement: given the assessment above, there were very minor concerns regarding confidence in evidence from this subtheme. Evidence here is most applicable to spina bifida and the majority of quotes came directly from children or adolescents.

##### Subtheme 4: Children and Adolescents Perceiving Themselves as Different from Their Peers; That They Do Not Fit in due to Physical Differences Limiting Activity Participation

This subtheme is supported by five studies. Children and adolescents discussed their negative self-perception and desire to feel normal and fit in with their peers. Many children and adolescents discussed feeling ashamed due to their disability and physical symptoms, such as incontinence or limited ambulation.

Example: 

*‘The school took me out of my comfort zone… I became aware that I’m different from some children who stared at me and made fun of my shaky walk’ (12 years, female)*

[26].

Uniqueness: 

This subtheme was supported by 20 quotes. Male participants (n = 5/20, 25%) stated that they did not want to be judged because of their disabilities and highlighted not having their own friends but rather playing with siblings’ friends. Female participants explained how they were made aware of their physical differences and made to feel excluded and that they did not fit in. Furthermore, female participants (n = 14/20, 70%) highlighted being ridiculed by peers and one participant stated having friendships on social media that did not materialise into physical friendships. Children aged 10–13 years (n = 9/20, 45%) primarily highlighted their awareness of their differences and the ridicule they experienced from peers and awareness of differences between themselves and their peers. Adolescents aged 14–18 years (n = 8/20, 40%) trended towards not wanting to explain physical limitations to others and fear of peers’ judgement. Adolescents had greater self-awareness and were reflective on their experiences in striving for acceptance of their physical limitations.

Quotes from parents (n = 4/20, 20%) primarily focused on promoting social inclusion for their children and opportunity for friendships. Conversely, the children’s main focus (n = 16/20, 80%) was on their fear of ridicule or rejection due to physical limitations.

CERQual Summary: 

The criteria rating for this subtheme was rated as follows: (a) methodological limitations—minor concerns, (b) coherence—minor concerns, (c) adequacy of data—minor concerns, and (d) relevance—minor concerns. Minor concerns regarding coherence in Gorzkowski et al. [25]. Unclear influence of researcher on research. All papers examined spina bifida (Supplementary File S4). Confidence statement: given the assessment above, there were minor concerns regarding confidence in evidence from this subtheme. The evidence may be most applicable to children with spina bifida.

##### Subtheme 5: Emotions of Anger, Fear, or Sadness towards Limited Participation in School Activities and Social Events

This subtheme is supported by six studies. Children or adolescents feeling excluded from school activities, sports, and outings due to their physical limitations. Desire to participate and enjoyment when included in physical activities. Often a lacking external or teacher facilitation, resulting in negative attitudes towards PA. All individuals were diagnosed with spina bifida.

Example: 

*‘I would like to join a public sports club. I love swimming and playing football with other children… I am very sad… It is difficult to reach these areas… I am not allowed to participate… I prefer to stay home’. (Boy, 16)*

[26].

*‘It sucked for a while; I was a pretty active person so the first 6–7 months kind of sucked’*

[80].

Uniqueness: 

This subtheme was represented by 10 participant quotes. Male participants (n = 4/10, 40%) identified their desire to participate in sports, such as football, and access being hindered by their teachers, causing sadness or frustration. One negative case was identified where a male participant explained that he was able to use the gym when imitating his friends. Female participants (n = 4/10, 40%) explained their fears or worries around participation due to their movement limitations.

All direct quotes from children (n = 8/10, 80%) focused on desire to participate and being either incorporated or excluded, depending on their teachers’ decisions to modify activities. Exclusion from PA consistently resulted in emotions of either sadness or frustration.

CERQual Summary: 

The criteria rating for this subtheme was rated as follows: (a) methodological limitations—minor concerns, (b) coherence—minor concerns, (c) adequacy of data—no /very minor concerns, and (d) relevance—no/very minor concerns. Four studies looking at spina bifida, one at SCI, and one at returning to PA post-spinal surgery. Very minor concerns. Concerns regarding coherence and the lack of representation in Gorzkowski et al. [25], and influence of researcher on the research. All other studies well represented and accounted for (Supplementary File S4). Confidence statement: given the assessment above there were very minor concerns regarding confidence in evidence from this subtheme. This subtheme is primarily suited to children and adolescent perceptions rather than parents.

##### Subtheme 6: Need to Adjust and Accept Spinal Pain or Disability and Its Influence on Participation in Sports, Exercise, and PA

This subtheme is supported by four studies. Participants desire for community awareness, adaptations, and acceptance of spinal pain or disability. Children and adolescents discussed their feelings towards their disability and for some the journey to accepting their condition. All participants discussed adapting their lifestyles as part of adjustment.

CERQual Summary: 

*‘I went through very many emotional rollercoasters to finally realise that I was not like every-one else and I had to accept that’ (14 years, female)*

[31].

Uniqueness: 

This subtheme was represented by 11 participant quotes. Male participants (n = 3/11, 27%) expressed a desire to join in with activities, problem-solve, and participate. Female participants (n = 3/11, 27%) expressed sadness, depression, and upset regarding their condition and the need to accept their physical condition and adapt to it.

Caregivers’ quotes (n = 5/11, 45%) expressed not accepting their children’s diagnosis and hiding their children. Caregivers also expressed a problematic lack of public awareness and acceptance. Conversely, child quotes (n = 5/11, 46%) described a journey to acceptance through depression and varying emotions. Children and adolescents described finding a constructive means to acceptance in sports, activities, and striving for independence.

CERQual Summary: 

The criteria rating for this subtheme was rated as follows: (a) methodological limitations—minor concerns, (b) coherence—no/very minor concerns, (c) adequacy of data—minor concerns, and (d) relevance—minor concerns. There was no evidence of negative cases. Data appear otherwise well saturated and adequate with good coherence and relevance. This subtheme is most applicable to those with spina bifida (Supplementary File S4). Confidence statement: given the assessment above there are minor concerns.

#### 3.5.3. Theme 3: Sociological Factors

This theme was identified according to Kamper, et al. [33] and defined as factors that encompass socioeconomic status, environmental influence, and cultural factors [79]. These may include friends, family, and relationships, as well as access to equipment and resources. Seven subthemes were identified within this theme.

Subthemes: 

##### Subtheme 7: Fear and Worry towards Participation in Sports, Exercise and PA; Fear towards Socialising in a Sports Group and Peer Rejection

This subtheme is supported by five papers. Fear and worries surrounding injury or getting hurt by others during participation in PA, sports, and exercise. Fears around lack of friendships or social exclusion in sports, activities, or public situations.

Example: 

*‘Of those killing, killing kids, girls, taking girls and grabbing them and killing taking the body parts. I don’t go outside anymore. I never go outside’ (13 years, female)*

[27].

Uniqueness: 

This subtheme was represented by nine participant quotes. Female participants (n = 4/9, 44%) exclusively expressed fear and worry about going outside and socialising with friends in public places due to danger from others or being targeted.

Child direct quotes (n = 7/9, 77%) examined fear and worry around physical dangers from others, fear of rejection from peers, and fears surrounding inability to function in adult life.

CERQual Summary: 

The criteria rating for this subtheme was rated as follows: (a) methodological limitations—minor concerns, (b) coherence—minor concerns, (c) adequacy of data—no/very minor concerns, and (d) relevance—minor concerns. Studies are well represented examining population of interest. Three of five studies examining spina bifida, one spinal-cord injury, and one post-operative spinal surgery. Some concerns regarding coherence and lack of representation in Gorzkowski et al. [25] (Supplementary File S4). Confidence statement: given the assessment above, there were minor concerns regarding confidence in evidence from this subtheme.

##### Subtheme 8: Dependence of Child on Caregivers and Impact on Routine (Work, Family, and Time)

This subtheme is supported by four studies. Some participants expressed a desire to be independent with mobility, ambulation, or a self-propelling wheelchair. Others discussed their reliance on parent or caregiver support. Desire to access and attend sports clubs and activities independently of their parents. All participants discussed concerns related to dependence on their parents or caregivers for support.

Example: 

*‘Managing university studies alongside a serious health condition and restrictions in mobility can be a daily struggle. I will not be able to study at university, or to work and get married. I’m afraid of what will happen to me if my mother is no longer able to care for me… who will help me in this miserable life?’ (14 years, male)*

[26].

Uniqueness: 

This subtheme was represented by 12 participant quotes. Male participants (n = 5/12, 42%) quotes focused on dependence on parents and requirement for assistance with mobility, toileting, and sports or physical activities. This resulted in both sadness and worry. One negative case of parents acting as problem-solvers and improvising to reduce dependence. Participants of an unknown gender (n = 6/12, 50%) focused on promoting self-efficacy and promoting independence, self-propelling wheelchair, or getting to clubs or activities independently.

Adolescents aged 14–18 years (n = 4/12, 33%) stated a desire and inability to participate in sports clubs due to the need for physical assistance. Adolescents stated both worries and a desire to complete university studies or work, achieve independence with toileting, and reduce reliance on parents or caregivers.

Direct quotes from children or adolescents (n = 9/12, 75%) rather than parents or caregivers centred around a desire to be independent and increase mobility and participation in sports and activities.

CERQual Summary: 

The criteria rating for this subtheme was as follows: (a) methodological limitations—minor concerns, (b) coherence—no/very minor concerns, (c) adequacy of data—no/very minor concerns, and (d) relevance—minor concerns. This subtheme is well represented across the four papers. Minor concerns regarding methodological quality and researcher influence. Data appear well saturated, however, with evidence of negative cases. All studies examine SB and no other paediatric spinal conditions examined (Supplementary File S4). Confidence statement: given the assessment above, there were minor concerns regarding confidence in evidence from this subtheme.

##### Subtheme 9: Negative Attitudes from Others and Lack of Social Acceptance; Experiencing Ridicule and Feeling Ashamed

Subtheme is supported by six papers. Participants discussed feeling negative emotions towards the differences between themselves and others. Hiding aspects of their disability, feeling ashamed, or being stigmatised.

Example: 

*‘The reason that I have not told my friends is that I see how they are now. Young people, and now I mean people my age, they are kind of, what can I say, immature, or they say certain things. They think that if a person is different, then that person is a freak, or something like that. That is why I have not dared (to tell them about having SB)’ (15 years, male)*

[32].

Uniqueness: 

This subtheme was represented by 16 participant quotes. Both male (n = 10/16, 63%) and female participants (n = 4/16, 25%) explained that they experienced ridicule and lacked social acceptance. Male participants discussed hiding aspects of their physical disability from their peers. One negative case was identified where a male participant explained that his friends and cousins were helpful and encouraging. Female participants discussed their peers perceiving them as different and lacking social acceptance.

Children aged 10–13 years (n = 4/16, 25%) stated that they felt out of place and different from their peers. The adolescents aged 14–18 years (n = 9/16, 56%) described not wanting to tell their friends about their incontinence issues or physical impairments to avoid judgement.

CERQual Summary: 

The criteria rating for this subtheme was as follows: (a) methodological limitations—minor concerns, (b) coherence—no/very minor concerns, (c) adequacy of data—no/very minor concerns, and (d) relevance—no/very minor concerns. Supported by six papers and results demonstrate very minor concerns. There was some lack of detail of the influence of the researcher on the research. Data were, otherwise, well represented; all papers examined spina bifida, but population of interest is across a variety of locations and populations (Supplementary File S4). Confidence statement: given the assessment above, there were minor concerns regarding confidence in evidence from this subtheme.

##### Subtheme 10: Friends Providing Needed Emotional Support and Role Models

This subtheme is supported by six papers. All participants discussed the positive impacts of friendships and feelings of acceptance with friends. Some participants discussed the positive influence this had on participation in PA, sports, and exercise.

Example: 

Three participants reported *‘many close friends’, ‘weekly sleepovers with friends’, and ‘a consistent group of close friends that she spent time with every weekend’*

[31].

Uniqueness: 

This subtheme was represented by 12 participant quotes. Both younger children aged 7–9 years (n = 6/12, 50%) and adolescents aged 14–18 years (n = 3/12, 25%) described having many close friends. Younger children described social acceptance as a facilitator of PA, sports, and exercise, whilst the adolescents described feeling encouraged by their friends.

One negative case was described by a female participants caregiver who stated that she was bullied and hit at school, and came home with a blue eye [27].

CERQual Summary: 

The criteria rating for this subtheme was as follows: (a) methodological limitations—minor concerns, (b) coherence—no/very minor concerns, (c) adequacy of data—no/very minor concerns, and (d) relevance—minor concerns. Supported by six studies and the results demonstrated minor concerns. There was some lack of clarity in coherence in Pfeiffer et al. [28] due to indirect participant quotes. Otherwise, data were well saturated with good adequacy. Minor concerns regarding relevance, one study examining achondroplasia and five spina bifida (Supplementary File S4). Confidence statement: given the assessment above, there are minor concerns regarding confidence in evidence from this subtheme.

##### Subtheme 11: Youth Dependent on Parents for Physical and Emotional Advice or Support

This subtheme is supported by seven papers. Parents and caregivers providing both physical support and facilitation, but also providing emotional support and encouragement. Helping their child pursue independence, function, and participation in sports, exercise, and physical activities.

Example: 

*‘I knew that I had to because my parents aren’t going to be there in Grade 1 and I just didn’t want everybody knowing and I didn’t want everybody to be involved in it. I’m just like okay, I’m gonna do it’ (9 years, female)*

[31].

Uniqueness: 

This subtheme was represented by 13 participant quotes. Male participants described needing parental support for mental well-being and problem-solving. Female participants described parental support regarding mental health, physical activities and exercise, and promoting independence.

Younger children aged 7–9 years (n = 4/13, 31%) described a reliance on parental support for physical assistance and parents being pivotal in developing a desire for independence. Children aged 10–13 years (n = 2/13, 15%) explained that their parents provided emotional support, aiding in continuing with their physical impairments and make the best of their situation. The adolescents aged 14–18 years (n = 4/13, 31%) primarily focused on the future and need to take a problem-solving approach, and some stated they had worries about independence.

Parents (n = 4/13, 31%) consistently expressed a desire for their children to be independent with self-care and, thereby, facilitating participation in PA, sports clubs, or exercise. Children/adolescents direct quotes (n = 9/13, 69%) stated that parents were pivotal in mental wellbeing, and encouraging PA and functioning.

CERQual Summary: 

The criteria rating for this subtheme was as follows: (a) methodological limitations—minor concerns, (b) coherence—no/very minor concerns, (c) adequacy of data—minor concerns, and (d) relevance—no/very minor concerns. Data were well saturated with seven papers examining this subtheme—one study examining achondroplasia, another post-operative paediatric spinal surgery, and the others spina bifida. There were no concerns with regards to coherence, adequacy, or relevance. There was some lack of information across all studies’ methodology regarding the influence of the researcher on the research (Supplementary File S4). Confidence statement: given the assessment above, there were very minor concerns regarding confidence in evidence from this subtheme.

##### Subtheme 12: Lacking Information or Support from Sports Counsellors, Local Clubs or Organisations Regarding PA

This subtheme is supported by four papers. Parents and caregivers consistently discussed requirement for further information and advice regarding opportunities for physical activities, sports, and promoting function; consistently discussing the need for greater awareness of spina bifida or spinal conditions, implications, and opportunities available.

Example: 

*‘A lot of things you have to find out yourself… I do miss that…I think, if you’re in a hospital, we visit the hospital regularly, that there should be…more information…and listening to what the child wants and I do miss that…they ask for example ‘how is it’, ‘yes everything goes well’ he (the child) says, well he always says everything goes well…but I think…you should ask ‘what else do you want, how is it going with playing sports, do you play sports’, it is always about what school do you go to and that’s that’ (Parent, child 8–18 years, unknown gender)*

[24].

Uniqueness: 

This subtheme was represented by 10 participant quotes. Parents (n = 4/10, 40%) consistently stated that the support or lack of support from professionals or teachers was pivotal in facilitating PA, sports, or exercise. Caregivers (n = 4/10, 40%) highlighted a lack of support from community programs. Caregivers stated a desire to raise social awareness and opportunities available for children and adolescents.

CERQual Summary: 

The criteria rating for this subtheme was as follows: (a) methodological limitations—minor concerns, (b) coherence—minor concerns, (c) adequacy of data—minor concerns, and (d) relevance—minor concerns. This subtheme is supported by four papers with minor concerns. Some lack of representation of participant quotes in Gorzkowski et al. [25]. There was no evidence of negative cases (Supplementary File S4). Confidence statement: given the assessment above, there were minor concerns regarding confidence in evidence from this subtheme.

##### Subtheme 13: Desire to Be Active and Increase Environmental Access

This subtheme is supported by four papers. Desire from children/adolescents or their parents about being independent within society. Desire for physical mobility independence, e.g., self-propelling wheelchair. Desire from participants and their parents or caregivers to be independent into adult life with work or studies.

Example: 

*‘Cause I don’t wanna be, like, in a wheelchair—it’s not fun to be in. Then I can do nothing, like, say (me) now I wanna go play soccer, then I can’t cause I’m in here. And then I can’t do what other children can do’ (13 years, male)*

[27].

Uniqueness: 

This subtheme was represented by 12 participant quotes. Both male (n = 5/12, 42%) and female (n = 4/12, 33%) participants had a desire to be active. Male participants stated wanting to be independent as similar to other able-bodied children as possible. Female participants highlighted their mobility impairments and their desire to be similar to their peers and join in with PA.

Children aged 10–13 years (n = 4/12, 33%) had a desire to play sports, to be out of wheelchair, and participate alongside their peers. Meanwhile, adolescents aged 14–18 years (n = 4/12, 33%) stated wanting to be dissociated from their wheelchair and to be independent at university or into adult life.

CERQual Summary: 

The criteria rating for this subtheme was as follows: (a) methodological limitations—minor concerns, (b) coherence—no/minor concerns, (c) adequacy of data—minor concerns, and (d) relevance—no/minor concerns. Supported by four papers. There was some lack of representation in populations other than spina bifida affecting the relevance of results. This subtheme is otherwise well represented and supported by participant quotes with good coherence. There was no evidence of negative cases influencing adequacy (Supplementary File S4). Confidence statement: given the assessment above, there were minor concerns regarding confidence in evidence from this subtheme. It is most applicable to individuals with spina bifida.

Certainty of evidence 

The JBICQR showed that further information was required for Pfeiffer et al. [28] and Wilson et al. [30]. All other studies included in the review were at low risk of bias (Supplementary File S1). The GRADE-CERQual assessment was performed for each subtheme and has identified minor concerns [68], therefore, we are able to have confidence in the evidence. For each subtheme any gaps in saturation or comments have been identified, alongside uniqueness with a summary for the GRADE-CERQual scoring to help identify how well understood each theme or subtheme is. For further information on the GRADE-CERQual summary scoring, please see Supplementary File S4.

## 4. Discussion

To the best of the authors’ knowledge, this is the first attempt at synthesising evidence detailing factors influencing exercise participation in paediatric spinal pain or spinal conditions, and identification of any trends in conditions, age, gender, or other demographics [80]. Findings describe factors influencing participation in sports, exercise, and PA in paediatric spinal pain and the differences between children and adolescents, male and female, and parents and caregivers. Review findings highlight the paucity of qualitative evidence in factors influencing participation in sports, exercise, and PA in relation to musculoskeletal conditions. The synthesis may be most relevant to those with neuromuscular conditions, particularly, spina bifida for which most of evidence is derived [24,26,27,29,31,32]. Evidence suggests that individuals with spina bifida have no relationship between attitudes and perceptions towards organised and non-organised PA [81]. Conversely, review findings along with other evidence suggests that sociological factors, such as being part of an organised group or team, positively influence participation [81].

The ICF-CY was chosen to help define PA, sports, and exercise for the purposes of this project [34]. Results demonstrated a wide spectrum of physical function with some participants discussing bladder and bowel control as a disability-related obstacle [24,25,26,31,32]. Meanwhile, other qualitative data focused on participation in sports clubs and physical education classes [25,26,29,30,31,32]. Therefore, this review, in accordance with other literature, adopted a partial implementation of the ICF-CY, allowing it to help us define PA, sports, and exercise, but using it alongside the biopsychosocial concept to help understand the nature of factors influencing participation [82]. Further longitudinal research is required regarding the relationship between patient quality of life and functioning, as well as further validation through patient and clinician experience more globally [39,83].

Data were analysed in accordance with the biopsychosocial model—biological, psychological, and social factors. Notwithstanding the overlapping nature of the biopsychosocial domains, sociological factors contributed the greatest proportion of subthemes. However, many clinicians tend to focus on biological factors whilst grouping together psychological and social factors [42]. This review demonstrates that, in order for the biopsychosocial model to be fully embraced, it is essential that sociological factors are not overlooked when understanding factors influencing participation in sports, exercise, and PA. Furthermore, that each of the biological, psychological, and sociological components are conceptually linked rather than regarded as separate entities [84]. The interaction between components is used as a predictor of individual outcomes rather than considering factors in isolation [42]. Furthermore, both the subtle realist stance and the biopsychosocial model enable a holistic understanding of factors influencing exercise to inform health-care practice and determine a reality for this patient demographic rather than an objective truth [40,85,86]. Although subtle realism does not allow absolute certainty, this paradigm reflects the nature of the question, namely, factors influencing sports, exercise, and PA participation, rather than researcher bias [87].

Contrary to popular belief [41], biological factors were the smallest proportion of factors influencing sports, exercise, and PA participation, with only two subthemes being identified. Subtheme 1 (challenges to PA, sports, or exercise participation resulting from biological aspects of physical condition), showed those aged 10–18 years have a desire to be active and overcome physical challenges, rather than be perceived as different. Children and adolescents have a higher self-efficacy regarding their physical ability compared to parents’ or caregivers’ perceptions of ability. There is a significant lack of literature examining factors influencing PA participation. However, one review examined two papers containing children and adolescents aged 9–25 years with neurological disabilities and suggests that participation in sports helps an individual redefine themselves, forgetting that they are unable to walk or have physical limitations [88]. Further evidence across a variety of physical disabilities and chronic diseases in those aged 10–19 years shows that increasing sports participation aids in improving or maintaining health whilst also increasing athletic competence [88,89]. This review was unique in examining paediatric spinal pain and offered insight into adolescents aged 14–18 years discussing their awareness of the physical challenges of exercise, whilst the children aged 10–13 years exhibited a higher self-efficacy despite their disability or spinal pain. Limited literature available in other groups suggests that the positive effects of overcoming disability gives a feeling of fun, success, competence, and opportunity for friendship [90]. This may help to explain why those who are already physically active have a greater desire to continue overcoming physical challenges with sports and exercise, despite increasing awareness of these challenges with increasing age. However, further research is required to understand the shift with age in self-efficacy and disability awareness. However, these biological factors manifesting as limitations are inextricably linked with psychological and social factors, notably, self-efficacy [40]. Although the factors influencing participation described are categorised as biological, it is arguable that their qualitative, rather than objective, nature strongly overlaps with psychological factors, as per the biopsychosocial model [40,42].

Subtheme 2 (bladder and bowel care and its impact on participation in PA, sports, and exercise) highlighted that younger children (8–9 years) strived for independence to complete their catheterisation autonomously to facilitate exercise participation independently of their parents. Adolescents (14–18 years) desired secrecy regarding their incontinence, and similar to subtheme 1, greater awareness of the difference between themselves and their peers whilst still having a desire to join their peers. Parents and caregivers of individuals aged 8–18 years highlighted a desire for their child to achieve independence with bladder and bowel care in order to facilitate their child participating in PA with their peers. This qualitative review has demonstrated that factors labelled as biological influences on participation are still heavily influenced by psychological factors, such as subjective perception of physical ability, rather than a pure objective measure. This is fitting with the literature highlighting that individual frame of reference regarding their disability and function will depend on whether they are participating in sports alongside able-bodied peers, or those with similar disability or pain [88]. Therefore, what an individual perceives as biologically limiting them is still heavily influenced by their psychology [88]. Furthermore, evidence suggests those with disabilities who are already participating in sports have higher reported athletic competence and self-efficacy regarding their physical ability than those who are not participating [89]. This has the potential to confound biological factors and the reader must understand the biopsychosocial continuum rather than each component of the biopsychosocial model being a distinct part [89]. Consideration of the individuals background and their current activity levels is vital in drawing conclusions regarding factors influencing exercise participation. Furthermore, this review highlights the importance of physiotherapists opening conversations with children and adolescents about perception, exercise participation, and individual background and beliefs.

Psychological factors related to the influence of thoughts and feelings or emotions on behavior [79]. When discussing subtheme 6 (the need to adjust and accept spinal pain or disability and its influence on participation in sports, exercise, and PA) all participants discussed adapting their lifestyles as part of adjusting and accepting their condition. Interestingly, the main differences here were between male and female participants. The males discussed a desire to join in with activities, problem-solve, and participate. This is consistent with one study looking at adults which suggested that typically men with disabilities have higher emotional intelligence and resilience compared to men without disabilities or women thereby making them more likely to respond positively towards PA, sports, and exercise [91]. Conversely, there is other literature to suggest that males are more likely than females to perceive themselves as having a disability and as different from others [92]. The question remains how this can be further understood by specific spinal conditions and whether the rate of participation between males and females has any correlation with self-perception. Conversely, female participants exhibited negative emotion sadness, depression, and upset regarding their limited physical ability whilst also expressing a need to accept and adapt. Young women with disabilities have reported feeling more depressed or dissatisfied with their body shape, thereby affecting their self-worth, however, further research is required to test this and the influence it has on rate of exercise uptake and types of exercise [93]. There appears to be very little literature examining the relationship between psychological factors, gender, and exercise uptake in children and adolescents with spinal pain. On study examined self-worth scores in paediatric disability and found no significant differences between genders [93], this was found to be consistent with a variety of other authors [94,95]. Further up-to-date literature is required to examine the differences in whether individuals are accepting of their condition and the impact on feelings, self-worth, and exercise participation between genders, especially in paediatric spinal pain and how this differs across spinal conditions.

The results show a trend towards sociological issues as primary influences regarding participation in exercise, sports, and PA. Subtheme 9 (negative attitudes from others and lack of social acceptance, experiencing ridicule, and feeling ashamed) highlighted a lack of social acceptance as a result of their disability negatively affecting their decision to participate in sports, exercise, or PA [24,26,27,29,31,32]. The younger children (10–13 years) discussed feeling out of place for fear of judgement from their peers and the older adolescents (14–18 years) discussed not wanting to tell their peers about issues such as incontinence or physical impairments. This fits with existing literature looking at adolescents with restricted mobility having strained relationships with peers but desiring acceptance [96]. Many physiotherapists tend to focus on biological and psychological factors when dealing with back pain but often neglect sociological factors [42]. Contrary to the results exhibited in subtheme 9, some social influences in youth with spina bifida have a positive influence on factors relating to sports, exercise, and PA participation such as self-efficacy, mental health and enjoyment of PA [29]. Research suggests that addressing sociological factors in adult spinal pain improves movement, function, and pain [97]. However, there still remains a lack of evidence regarding what happens in clinical practice and how best to utilise these sociological factors to inform the physiotherapeutic care and encourage PA participation in under 18s with spinal conditions or spinal pain [42].

For this review participants were divided into two groups: children aged 0–13 years and adolescents aged 14–18 years. This was based on literature highlighting the shift in social maturity during teenage years as well as children being transitioned to adult services after the age of 14 years [69,73,74]. The narrative portrayed by the adolescents over 14 years offered a different perspective to the children, with a greater self-awareness and a critical insight. Furthermore, the younger children commonly talked about things with a parental relationship perspective, however, the older children often expressed a desire for independence of contextualised their thoughts or feelings in relation to moving towards adulthood. The results have inevitable variability in adolescent experience depending on the culture, location, and type of health-care received will influence individual experience and factors influencing choice to participate in exercise [98]. Further research on the implications of age of transition for spinal pain and cultural influence, and uniqueness of factors influencing exercise participation across different specific ages rather than groups of individuals would be of benefit in informing how best to engage with these individuals. In the absence of age specific information this paper examined groups of individuals to draw meaningful conclusions and implications for clinical practice. The choice of subtle realism enables subjective perceptions and observations to be considered and despite not having absolute certainty, we can draw meaningful conclusions and aid in improving health-care research [87].

### 4.1. Strengths and Limitations

This review had multiple strengths, including results being appraised for quality in several ways: firstly, the methodology for this review was based on the widely used and accepted seven stages of meta-ethnography [63]; secondly, the PRISMA checklist was used to ensure that all relevant information was reported to improve transparency [59]; thirdly, individual studies were quality assessed using the JBICQR [64,67]; and, finally, between-study quality analysis was performed using GRADE-CERQUAL [68]. GRADE-CERQUAL was chosen as it allows a summary of qualitative findings and overall assessment across multiple papers whilst examining the extent to which the review finding represents the phenomenon of interest [99]. The JBICQR was chosen due to its associated congruity and coherence [65]. From the studies identified some concerns were identified in Willson et al. [30] surrounding representation of participants voices. A systematic review and meta-ethnography were chosen due to their value in generating models, or theories of behaviour and experiences, and re-interpreting significance across multiple studies [100].

Limitations include the content of the primary research used to form the review. Each of the studies lacked information regarding the influence of the researcher on the research, and one of the studies lacked information around philosophical perspective and methodology [31]. One of the studies lacked information around the researchers’ cultural or theoretical stance [28], and five of the studies lacked information on both these components [25,26,27,30,32]. Please see Supplementary File S1 for further information. A further limitation related to the identification of themes and subthemes. Although the majority of subthemes identified as sociological factors, there is an unavoidable overlap between biological and psychological influence and back pain outcomes [42]. Therefore, it is vital to understand the themes and subthemes identified are conceptually linked to each other through the biopsychosocial model rather than separate entities [84]. A second model used was the ICF-CY to help identify the meaning of PA, sports, and exercise. However, the ICF-CY has been shown to have disadvantages related to limitations in content, its complexity, and time required for application [82]. Furthermore, the ICF-CY is inherently disadvantageous in tis nature of purely assessing function without due regard to the individual [35]. Finally, this study was unique in its synthesis of factors influencing exercise participation in adolescent spinal pain by demographic groups. However, further research is required to evaluate these factors within specific musculoskeletal conditions, such as AIS, and determine if there are any further differences by gender or age within each condition.

### 4.2. Clinical Implications

Physiotherapists and health-care professionals must engage in conversation with children and adolescents regarding factors influencing PA participation and ensure that these factors are considered in the rehabilitation goal. Social acceptance and self-efficacy are important considerations in exercise participation, self-management, and a healthy active lifestyle. Physiotherapists and those caring for paediatric patients experiencing spinal pain or with spinal conditions ought to consider the role of group exercise and social acceptance when encouraging the benefits and purpose of PA, sports, and exercise. Adolescents (>14 years) typically focus on independence and secrecy regarding their condition, which may influence styles and types of exercises prescribed when encouraging activity participation particularly in group contexts. Further clinical research is required examining specific paediatric spinal conditions, such as AIS, and whether there are variations in factors influencing exercise participation by specific condition.

## 5. Conclusions

There is a substantial lack of qualitative data in paediatric and adolescent musculoskeletal spinal pain. Factors influencing participation in exercise, sports, and PA are complex and varied with a mixture of biological, psychological, and sociological factors overlapping. This review highlights that sociological factors are key influencers of participation in sports, exercise, and PA, and, furthermore, that younger children aged up to 13 years tended to identify themselves as different from their peers and needing parental support. Adolescents aged 14 to 18 years focused on gaining independence, a desire for secrecy regarding their condition, and the impact of their condition, on their adult lives.

## Figures and Tables

**Table 1:** SPIDER search strategy.

Sample:	Studies were eligible if they included: (a) children or adolescents aged 18 years or under, (b) experiencing thoracic or (c) lumbar spinal pain, or with any diagnosed spinal condition. Studies were excluded when there was a pure focus on the experience of neck pain.
Phenomena of interest:	Studies had to report on the effect of thoracic or lumbar diagnosed spinal condition or spinal pain on physical function. Studies were required to include qualitative data from participants who were aiming to participate in, or who were participating in, any kind of physical functioning, PA, exercise, or sports. Studies examining participation in PA as part of physiotherapy and rehabilitation following medical interventions were included, provided factors influencing participation were examined.
Design:	Types of qualitative data were required for inclusion. The different types included (but not limited to): ethnography, descriptive approaches, types of phenomenology, patient narratives, case studies, types of grounded theory, and action research. Studies were included where there was a clear identification, collection, and analysis of qualitative data.
Evaluation:	Studies had to include qualitative factors influencing participation in exercise, sports, and PA. Qualitative interviews, field notes, open questions, or focus groups that identified data surrounding PA, exercise, or sports were also included. Studies that only contained quantitative data, or that did not identify patient experiences, beliefs, or perceptions, were excluded.
Research Type:	Qualitative studies and mixed-methods studies were included. Conference proceedings, abstracts only, editorials, and opinions were excluded.
Other:	Studies where the language was not translatable into English were excluded. Studies requiring translation had to be translated by two authors independently. Both authors had to agree that the clarity and language was clear and without substantial error. Studies where the full text was unavailable from the inter-library loan system were excluded.

**Table 2:** Search strategy.

1	‘adolescen *’ OR ‘child *’ OR ‘teen *’ OR ‘youth’ OR ‘young person’ OR ‘juvenile *’
2	‘back’ OR ‘spinal’ OR ‘thoracic’
3	‘barrier *’ OR ‘facilitator *’ OR ‘reason *’ OR ‘feeling *’ OR ‘belief *’ OR ‘obstacle *’ OR ‘challenge *’
4	‘exercise *’ OR ‘physical activit *’ OR ‘sport *’ OR ‘physical *’ OR ‘activ *’
5	‘questionnaire *’ OR ‘focus group *’ OR ‘interview *’
6	‘participation’ OR ‘quality of life’ OR ‘QOL’
7	‘qualitative’ OR ‘mixed method *’ OR ‘narrative’ OR ‘grounded theory’ OR ‘phenomenology’ OR ‘ethnography’ OR ‘action research’ OR ‘case studies’
8	1 AND 2 AND 3 AND 4 AND 5 AND 6 AND 7

**Table 4:** Synthesis of the results.

Theme 1: Biological Factors	Theme 2: Psychological Factors	Theme 3: Sociological Factors
Challenges to PA, sports, or exercise participation resulting from biological aspects of physical condition.	Feelings of struggle and needing for physical assistance from others. Desire for independence when participating in sports, exercise, and PA.	Fear and worry towards participation in sports, exercise, and PA. Fear towards socialising in a sports group and peer rejection.
Bladder and bowel care and its impact on participation in PA, sports, and exercise.	Children and adolescents perceiving themselves as different from their peers, and that they do not fit in.	Dependence of child on caregivers and impact on routine (work, family, and time).
	Emotions of anger, fear or sadness towards limited participation in school activities and social events.	Negative attitudes from others and lack of social acceptance. Experiencing ridicule and feeling ashamed.
	Need to adjust and accept spinal pain or disability and its influence on participation in sports, exercise, and PA.	Friends providing needed emotional support and role models.
		Youth dependent on parents for physical and emotional advice or support.
		Lacking information or support from sports counsellors, local clubs or organisations regarding PA.
		Desire to be active and increase environmental access.

**Figure A1:**
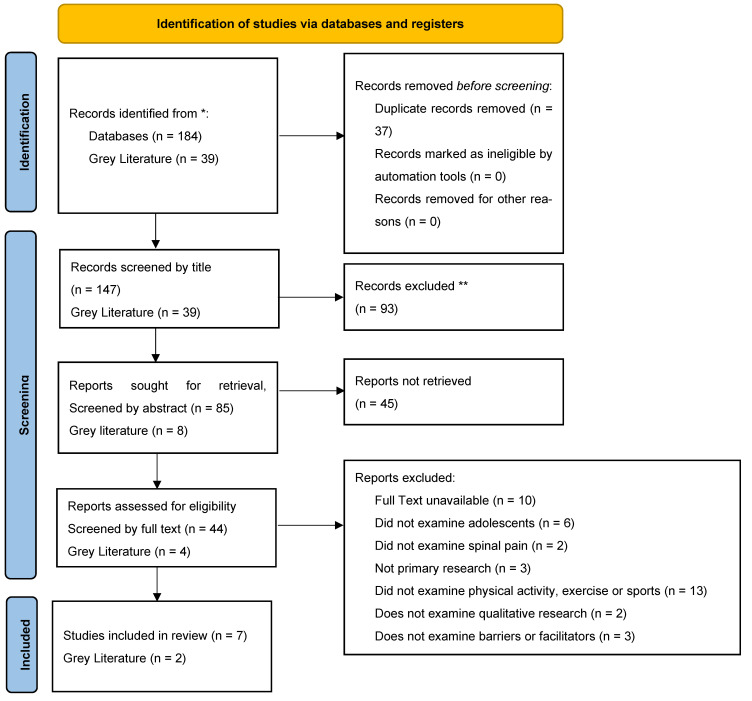
* Consider, if feasible to do so, reporting the number of records identified from each database or register searched [rather than the total number across all databases/registers]. ** If automation tools were used, indicate how many records were excluded by a human and how many were excluded by automation tools. For more information, visit: http://www.prisma-statement.org/ (URL accessed on 18 November 2021) [59].

## Data Availability

The data presented in this study are available upon reasonable request from the corresponding author.

## Supplementary Materials

The following supporting information can be downloaded at: https://www.mdpi.com/article/10.3390/bs13060486/s1. Supplementary File S1: JBICQR Checklist, Supplementary File S2: Participant Quotes, Supplementary File S3: Audit Trail, Supplementary File S4: CERQual Summary.

## References

[1] Wu A., March L., Zheng X., Huang J., Wang X., Zhao J., Blyth F.M., Smith E., Buchbinder R., Hoy D. (2020). Global low back pain prevalence and years lived with disability from 1990 to 2017: Estimates from the Global Burden of Disease Study 2017. Ann. Transl. Med..

[2] Jones G.T., Macfarlane G.J. (2005). Epidemiology of low back pain in children and adolescents. Arch. Dis. Child..

[3] Vigdal Ø.N., Storheim K., Munk Killingmo R., Småstuen M.C., Grotle M. (2021). Characteristics of older adults with back pain associated with choice of first primary care provider: A cross-sectional analysis from the BACE-N cohort study. BMJ Open.

[4] Hébert J.J. (2022). Spinal pain in childhood: Prevalence, trajectories, and diagnoses in children 6 to 17 years of age. Eur. J. Pediatr..

[5] Coenen P. (2017). Trajectories of Low Back Pain From Adolescence to Young Adulthood. Arthritis Care Res..

[6] Briggs A.M., Smith A.J., Straker L.M., Bragge P. (2009). Thoracic spine pain in the general population: Prevalence, incidence and associated factors in children, adolescents and adults. A systematic review. BMC Musculoskelet. Disord..

[7] Romano M., Minozzi S., Bettany-Saltikov J., Zaina F., Chockalingam N., Kotwicki T., Maier-Hennes A., Negrini S. (2012). Exercises for adolescent idiopathic scoliosis. Cochrane Database Syst. Rev..

[8] Alshami A.M. (2015). Prevalence of spinal disorders and their relationships with age and gender. Saudi Med. J..

[9] Gopinathan N.R., Viswanathan V.K., Crawford A.H. (2018). Cervical Spine Evaluation in Pediatric Trauma: A Review and an Update of Current Concepts. Indian J. Orthop..

[10] Fares J., Fares M.Y., Fares Y. (2017). Musculoskeletal neck pain in children and adolescents: Risk factors and complications. Surg. Neurol. Int..

[11] NHS (2021). Physical Activity Guidelines for Children and Young People. https://www.nhs.uk/live-well/exercise/exercise-guidelines/physical-activity-guidelines-children-and-young-people/.

[12] Calvo-Muñoz I., Gómez-Conesa A., Sánchez-Meca J. (2013). Physical therapy treatments for low back pain in children and adolescents: A meta-analysis. BMC Musculoskelet. Disord..

[13] Hayden J.A., Ellis J., Ogilvie R., Malmivaara A., van Tulder M.W. (2021). Exercise therapy for chronic low back pain. Cochrane Database Syst. Rev..

[14] Geneen L.J., Moore R.A., Clarke C., Martin D., Colvin L.A., Smith B.H. (2017). Physical activity and exercise for chronic pain in adults: An overview of Cochrane Reviews. Cochrane Database Syst. Rev..

[15] Mueller S., Mueller J., Stoll J., Prieske O., Cassel M., Mayer F. (2016). Incidence of back pain in adolescent athletes: A prospective study. BMC Sport. Sci. Med. Rehabil..

[16] Green B.N., Johnson C., Moreau W. (2009). Is physical activity contraindicated for individuals with scoliosis? A systematic literature review. J. Chiropr. Med..

[17] Frosch M., Leinwather S., Bielack S., Blödt S., Dirksen U., Dobe M., Geiger F., Häfner R., Höfel L., Hübner-Möhler B. (2022). Treatment of Unspecific Back Pain in Children and Adolescents: Results of an Evidence-Based Interdisciplinary Guideline. Children.

[18] Gov.UK New Physical Activity Guidelines 25 July 2011. https://www.gov.uk/government/news/new-physical-activity-guidelines.

[19] NICE (2016). Low Back Pain and Sciatica in Cver 16s: Assessment and Management. https://www.nice.org.uk/guidance/ng59/chapter/Recommendations.

[20] Michaleff Z.A., Kamper S.J., Maher C.G., Evans R., Broderick C., Henschke N. (2014). Low back pain in children and adolescents: A systematic review and meta-analysis evaluating the effectiveness of conservative interventions. Eur. Spine J..

[21] Motyer G.S., Kiely P.J., Fitzgerald A. (2021). Adolescents’ Experiences of Idiopathic Scoliosis in the Presurgical Period: A Qualitative Study. J. Pediatr. Psychol..

[22] Negrini S., Donzelli S., Aulisa A.G., Czaprowski D., Schreiber S., De Mauroy J.C., Diers H., Grivas T.B., Knott P., Kotwicki T. (2018). 2016 SOSORT guidelines: Orthopaedic and rehabilitation treatment of idiopathic scoliosis during growth. Scoliosis Spinal Disord..

[23] Joergensen A.C., Strandberg-Larsen K., Andersen P.K., Hestbaek L., Andersen A.N. (2021). Spinal pain in pre-adolescence and the relation with screen time and physical activity behavior. BMC Musculoskelet. Disord..

[24] Bloemen M.A., Verschuren O., van Mechelen C., Borst H.E., de Leeuw A.J., van der Hoef M., de Groot J.F. (2015). Personal and environmental factors to consider when aiming to improve participation in physical activity in children with Spina Bifida: A qualitative study. BMC Neurol..

[25] Gorzkowski J., Kelly E.H., Klaas S.J., Vogel L.C. (2011). Obstacles to community participation among youth with spinal cord injury. J. Spinal Cord Med..

[26] Nahal M.S., Axelsson A.B., Imam A., Wigert H. (2019). Palestinian children’s narratives about living with spina bifida: Stigma, vulnerability, and social exclusion. Child Care Health Dev..

[27] Page D.T., Coetzee B.J. (2021). South African adolescents living with spina bifida: Contributors and hindrances to well-being. Disabil. Rehabil..

[28] Pfeiffer K.M., Brod M., Smith A., Ota S., Charlton R.W., Viuff D. (2021). Functioning and well-being in older children and adolescents with achondroplasia: A qualitative study. Am. J. Med. Genet. Part A.

[29] Volfson Z., Arbour-Nicitopoulos K.P., McPherson A.C., Tomasone J.R., Faulkner G.E. (2020). Examining factors of physical activity participation in youth with spina bifida using the Theoretical Domains Framework. Disabil. Health J..

[30] Willson L.R., Klootwyk M., Rogers L.G., Sasseville C., Shearer K., Southon S. (2021). Timelines for returning to physical activity following pediatric spinal surgery: Recommendations from the literature and preliminary data. BMC Res. Notes.

[31] Fischer N. (2015). A qualitative exploration of the experiences of children with spina bifida and their parents around incontinence and social participation. Child Care Health Dev..

[32] Strömfors L., Wilhelmsson S., Falk L., Höst G.E. (2017). Experiences among children and adolescents of living with spina bifida and their visions of the future. Disabil. Rehabil..

[33] Kamper S.J., Apeldoorn A.T., Chiarotto A., Smeets R.J.E.M., Ostelo R.W.J.G., Guzman J., van Tulder M.W. (2015). Multidisciplinary biopsychosocial rehabilitation for chronic low back pain: Cochrane systematic review and meta-analysis. BMJ Br. Med. J..

[34] World Health Organization (2007). International Classification of Functioning, Disability and Health: Children and Youth Version: ICF-CY.

[35] Ptyushkin P., Vidmar G., Burger H., Marinček Č., Escorpizo R. (2011). The International Classification of Functioning, Disability and Health (ICF) in vocational rehabilitation and disability assessment in Slovenia: State of law and users’ perspective. Disabil. Rehabil..

[36] Hwang A.-W., Yen C.-F., Liou T.-H., Bedell G., Granlund M., Teng S.-W., Chang K.-H., Chi W.-C., Liao H.-F. (2015). Development and validation of the ICF-CY-Based Functioning Scale of the Disability Evaluation System—Child Version in Taiwan. J. Formos. Med. Assoc..

[37] Sanches-Ferreira M., Simeonsson R.J., Silveira-Maia M., Alves S., Tavares A., Pinheiro S. (2013). Portugal’s special education law: Implementing the International Classification of Functioning, Disability and Health in policy and practice. Disabil. Rehabil..

[38] De Polo G., Pradal M., Bortolot S., Buffoni M., Martinuzzi A. (2009). Children with disability at school: The application of ICF-CY in the Veneto region. Disabil. Rehabil..

[39] McDougall J., Wright V., Schmidt J., Miller L., Lowry K. (2011). Applying the ICF framework to study changes in quality-of-life for youth with chronic conditions. Dev. Neurorehabil..

[40] Borrell-Carrió F., Suchman A.L., Epstein R.M. (2004). The biopsychosocial model 25 years later: Principles, practice, and scientific inquiry. Ann. Fam. Med..

[41] Engel G.L. (1977). The need for a new medical model: A challenge for biomedicine. Science.

[42] Mescouto K., Olson R.E., Hodges P.W., Setchell J. (2020). A critical review of the biopsychosocial model of low back pain care: Time for a new approach?. Disabil. Rehabil..

[43] Hill J.J. (2016). Encouraging healthy spine habits to prevent low back pain in children: An observational study of adherence to exercise. Physiotherapy.

[44] Holopainen R., Simpson P., Piirainen A., Karppinen J., Schütze R., Smith A., O’Sullivan P., Kent P. (2020). Physiotherapists’ perceptions of learning and implementing a biopsychosocial intervention to treat musculoskeletal pain conditions: A systematic review and metasynthesis of qualitative studies. PAIN.

[45] Kimiecik J.C., Horn T.S., Shurin C.S. (1996). Relationships among Children’s Beliefs, Perceptions of Their Parents’ Beliefs, and Their Moderate-to-Vigorous Physical Activity. Res. Q. Exerc. Sport.

[46] Slade S.C., Patel S., Underwood M., Keating J.L. (2014). What Are Patient Beliefs and Perceptions About Exercise for Nonspecific Chronic Low Back Pain? A Systematic Review of Qualitative Studies. Clin. J. Pain.

[47] Sattar R., Lawton R., Panagioti M., Johnson J. (2021). Meta-ethnography in healthcare research: A guide to using a meta-ethnographic approach for literature synthesis. BMC Health Serv. Res..

[48] Munn Z., Stern C., Aromataris E., Lockwood C., Jordan Z. (2018). What kind of systematic review should I conduct? A proposed typology and guidance for systematic reviewers in the medical and health sciences. BMC Med. Res. Methodol..

[49] Noblit G.W., Hare R.D., Hare R.D. (1988). Meta-Ethnography: Synthesizing Qualitative Studies.

[50] France E.F., Cunningham M., Ring N., Uny I., Duncan E.A.S., Jepson R.G. (2019). Improving reporting of meta-ethnography: The eMERGe reporting guidance. BMC Med. Res. Methodol..

[51] Young M.E., Ryan A. (2020). Postpositivism in Health Professions Education Scholarship. Acad. Med..

[52] Smith B. (2018). Generalizability in qualitative research: Misunderstandings, opportunities and recommendations for the sport and exercise sciences. Qual. Res. Sport Exerc. Health.

[53] Clark A.M. (1998). The qualitative-quantitative debate: Moving from positivism and confrontation to post-positivism and reconciliation. J. Adv. Nurs..

[54] NIHR (2023). PROSPERO International Prospective Register of Systematic Reviews. https://www.crd.york.ac.uk/prospero/.

[55] France E.F., Uny I., Ring N., Turley R.L., Maxwell M., Duncan E.A.S., Jepson R.G., Roberts R.J., Noyes J. (2019). A methodological systematic review of meta-ethnography conduct to articulate the complex analytical phases. BMC Med. Res. Methodol..

[56] Aromataris E., Munn Z. (2020). JBI Manual for Evidence Synthesis: JBI. https://jbi-global-wiki.refined.site/space/MANUAL.

[57] McGowan J., Sampson M., Salzwedel D.M., Cogo E., Foerster V., Lefebvre C. (2016). PRESS Peer Review of Electronic Search Strategies: 2015 Guideline Statement. J. Clin. Epidemiol..

[58] Bramer W. (2017). Updating search strategies for systematic reviews using EndNote. J. Med. Libr. Assoc..

[59] Page M.J., McKenzie J.E., Bossuyt P.M., Boutron I., Hoffmann T.C., Mulrow C.D., Shamseer L., Tetzlaff J.M., Akl E.A., Brennan S.E. (2021). The PRISMA 2020 statement: An updated guideline for reporting systematic reviews. BMJ.

[60] Cooke A., Smith D., Booth A. (2012). Beyond PICO: The SPIDER Tool for Qualitative Evidence Synthesis. Qual. Health Res..

[61] Porritt K., Gomersall J., Lockwood C. (2014). JBI’s Systematic Reviews: Study Selection and Critical Appraisal. Am. J. Nurs..

[62] Noblit G., Hare D. (1988). Meta-Ethnography: Synthesiszing Qualitative Studies.

[63] France E.F., Ring N., Thomas R., Noyes J., Maxwell M., Jepson R. (2014). A methodological systematic review of what’s wrong with meta-ethnography reporting. BMC Med. Res. Methodol..

[64] JBI (2017). The Joanna Briggs Institute Critical Appraisal Tools for Use in JBI Systematic Reviews Checklist for Qualitative Research. Critical Appraisal Checklist for Qualitative Research [Internet]. https://jbi.global/sites/default/files/2019-05/JBI_Critical_Appraisal-Checklist_for_Qualitative_Research2017_0.pdf.

[65] Hannes K. (2010). A Comparative Analysis of Three Online Appraisal Instruments’ Ability to Assess Validity in Qualitative Research. Qual. Health Res..

[66] Thomas J., Harden A. (2008). Methods for the thematic synthesis of qualitative research in systematic reviews. BMC Med. Res. Methodol..

[67] JBI Critical Appraisal Checklist for Qualitative Research 2020. https://jbi.global/critical-appraisal-tools.

[68] Lewin S., Booth A., Glenton C., Munthe-Kaas H., Rashidian A., Wainwright M., Bohren M.A., Tunçalp Ö., Colvin C.J., Garside R. (2018). Applying GRADE-CERQual to qualitative evidence synthesis findings: Introduction to the series. Implement. Sci..

[69] Care Quality Commission (2014). From the Pond into the Sea: Children’s Transition to Adult Health Services [PDF]. Gallowgate. https://www.cqc.org.uk/sites/default/files/CQC_Transition%20Report.pdf.

[70] Jarvis S., Richardson G., Flemming K., Fraser L. (2021). Estimation of age of transition from paediatric to adult healthcare for young people with long term conditions using linked routinely collected healthcare data. Int. J. Popul. Data Sci..

[71] Colver A., Rapley T., Parr J.R., McConachie H., Dovey-Pearce G., Couteur A.L., McDonagh J.E., Bennett C., Maniatopoulos G., Pearce M.S. (2020). Facilitating transition of young people with long-term health conditions from children’s to adults’ healthcare services—Implications of a 5-year research programme. Clin. Med..

[72] Narla N.P., Ratner L., Bastos F.V., Owusu S.A., Osei-Bonsu A., Russ C.M. (2021). Paediatric to adult healthcare transition in resource-limited settings: A narrative review. BMJ Paediatr. Open.

[73] World Health Organisation (2022). Adolescent Health. https://www.who.int/health-topics/adolescent-health#tab=tab_1.

[74] World Health Organisation Adolescence: A Period Needing Special Attention 2014. https://apps.who.int/adolescent/second-decade/section2/page1/recognizing-adolescence.html.

[75] United Nations Convention on the Rights of the Child. HUMAN RIGHTS [Internet]. 1989; CHAPTER IV(27531). https://treaties.un.org/doc/Treaties/1990/09/19900902%2003-14%20AM/Ch_IV_11p.pdf.

[76] Zimmer L. (2006). Qualitative meta-synthesis: A question of dialoguing with texts. J. Adv. Nurs..

[77] Wong G., Greenhalgh T., Westhorp G., Buckingham J., Pawson R. (2013). RAMESES publication standards: Realist syntheses. BMC Med..

[78] Page M.J., McKenzie J.E., Higgins J.P.T. (2018). Tools for assessing risk of reporting biases in studies and syntheses of studies: A systematic review. BMJ Open.

[79] Vögele C., Wright J.D. (2015). Behavioral Medicine. International Encyclopedia of the Social & Behavioral Sciences.

[80] McKenzie G., Willis C., Shields N. (2021). Barriers and facilitators of physical activity participation for young people and adults with childhood-onset physical disability: A mixed methods systematic review. Dev. Med. Child Neurol..

[81] Marques A., Maldonado I., Peralta M., Santos S. (2015). Exploring psychosocial correlates of physical activity among children and adolescents with spina bifida. Disabil. Health J..

[82] Jacob T. (2013). The implementation of the ICF among Israeli rehabilitation centers—The case of physical therapy. Physiother. Theory Pract..

[83] Karlsson E., Gustafsson J. (2022). Validation of the International Classification of Functioning, Disability and Health (ICF) core sets from 2001 to 2019—A scoping review. Disabil. Rehabil..

[84] Suls J., Rothman A. (2004). Evolution of the Biopsychosocial Model: Prospects and Challenges for Health Psychology. Health Psychol..

[85] Mays N., Pope C. (2000). Qualitative Research in Health Care: Assessing Quality in Qualitative Research. BMJ.

[86] Carpenter C. (1997). Conducting qualitative research in physiotherapy: A methodological example. Physiotherapy.

[87] Duncan E.A.S., Nicol M.M. (2004). Subtle Realism and Occupational Therapy: An Alternative Approach to Knowledge Generation and Evaluation. Br. J. Occup. Ther..

[88] Sahlin K.B., Lexell J. (2015). Impact of Organized Sports on Activity, Participation, and Quality of Life in People With Neurologic Disabilities. PM R..

[89] Te Velde S.J., Lankhorst K., Zwinkels M., Verschuren O., Takken T., de Groot J., HAYS study group (2018). Associations of sport participation with self-perception, exercise self-efficacy and quality of life among children and adolescents with a physical disability or chronic disease—A cross-sectional study. Sports Med. Open.

[90] Shields N., Synnot A. (2016). Perceived barriers and facilitators to participation in physical activity for children with disability: A qualitative study. BMC Pediatr..

[91] Díaz M.G., García M.J. (2018). Emotional intelligence, resilience and self-esteem in disabled and non-disabled people. Enfermería Glob..

[92] LoBianco A.F., Sheppard-Jones K. (2007). Perceptions of disability as related to medical and social factors. J. Appl. Soc. Psychol..

[93] Antle B.J. (2004). Factors associated with self-worth in young people with physical disabilities. Health Soc. Work.

[94] King G.A., Shultz I.Z., Steel K., Gilpin M., Cathers T. (1993). Self-Evaluation and Self-Concept of Adolescents With Physical Disabilities. Am. J. Occup. Ther..

[95] Stevens S.E., Steele C.A., Jutai J.W., Kalnins I.V., Bortolussi J.A., Biggar W.D. (1996). Adolescents with physical disabilities: Some psychosocial aspects of health. J. Adolesc. Health.

[96] Lisa Skär R.N. (2003). Peer and adult relationships of adolescents with disabilities. J. Adolesc..

[97] Becker A., Angerer P., Weber J., Müller A. (2020). The prevention of musculoskeletal complaints: Long-term effect of a work-related psychosocial coaching intervention compared to physiotherapy alone—A randomized controlled trial. Int. Arch. Occup. Environ. Health.

[98] McManus Holroyd A.E. (2007). Interpretive Hermeneutic Phenomenology: Clarifying Understanding. Indo-Pac. J. Phenomenol..

[99] Lewin S., Bohren M., Rashidian A., Munthe-Kaas H., Glenton C., Colvin C.J., Garside R., Noyes J., Booth A., Tunçalp Ö. (2018). Applying GRADE-CERQual to qualitative evidence synthesis findings—Paper 2: How to make an overall CERQual assessment of confidence and create a Summary of Qualitative Findings table. Implement. Sci..

[100] Atkins S., Lewin S., Smith H., Engel M., Fretheim A., Volmink J. (2008). Conducting a meta-ethnography of qualitative literature: Lessons learnt. BMC Med. Res. Methodol..

