# Supplementary material for: Factors Influencing Participation in Physical Activity, Sports, and Exercise in Children and Adolescents with Spinal Pain or Spinal Conditions: A Systematic Review and Meta-Ethnography
Source: Behav Sci (Basel). 2023 Jun 9;13(6):486. doi: 10.3390/bs13060486 (PMC10295783; doi:10.3390/bs13060486)
Supplement: Supplementary file 1 [file behavsci-13-00486-s001.zip › behavsci-2331584-supplementary.pdf]

Supplementary File S1: **JBICQR Checklist**

1] JBI CRITICAL APPRAISAL CHECKLIST FOR QUALITATIVE RESEARCH

Reviewer S.Magowan **Date** 17/05/22

Author Bloemen MAT, Verschuren O, van Mechelen C, Borst HE, de Leeuw AJ, van der Hoef M, et al. Personal and environmental factors to consider when aiming to improve participation in physical activity in children with Spina Bifida: a qualitative study. BMC neurology. 2015;15:11.

	Yes	No	Unclear	Not applicable
1. Is there congruity between the stated philosophical perspective and the research methodology?	X	☐	☐	☐
2. Is there congruity between the research methodology and the research question or objectives?	X	☐	☐	☐
3. Is there congruity between the research methodology and the methods used to collect data?	X	☐	☐	☐
4. Is there congruity between the research methodology and the representation and analysis of data?	X	☐	☐	☐
5. Is there congruity between the research methodology and the interpretation of results?	X	☐	☐	☐
6. Is there a statement locating the researcher culturally or theoretically?	X	☐	☐	☐
7. Is the influence of the researcher on the research, and vice- versa, addressed?	☐	X	☐	☐
8. Are participants, and their voices, adequately represented?	X	☐	☐	☐
9. Is the research ethical according to current criteria or, for recent studies, and is there evidence of ethical approval by an appropriate body?	X	☐	☐	☐
10. Do the conclusions drawn in the research report flow from the analysis, or interpretation, of the data?	X	☐	☐	☐

Overall appraisal: Include X Exclude ☐ Seek further info ☐

Comments [Including reason for exclusion]

2] JBI CRITICAL APPRAISAL CHECKLIST FOR QUALITATIVE RESEARCH

Reviewer S.Magowan

Date 17/05/22

Author Gorzkowski J, Kelly EH, Klaas SJ, Vogel LC. Obstacles to community participation among youth with spinal cord injury. The journal of spinal cord medicine. 2011;34[6]:576-85.

	Yes	No	Unclear	Not applicable
1. Is there congruity between the stated philosophical perspective and the research methodology?	☐	☐	X	☐
2. Is there congruity between the research methodology and the research question or objectives?	X	☐	☐	☐
3. Is there congruity between the research methodology and the methods used to collect data?	X	☐	☐	☐
4. Is there congruity between the research methodology and the representation and analysis of data?	X	☐	☐	☐
5. Is there congruity between the research methodology and the interpretation of results?	X	☐	☐	☐
6. Is there a statement locating the researcher culturally or theoretically?	☐	X	☐	☐
7. Is the influence of the researcher on the research, and vice- versa, addressed?	☐	X	☐	☐
8. Are participants, and their voices, adequately represented?	X	☐	☐	☐
9. Is the research ethical according to current criteria or, for recent studies, and is there evidence of ethical approval by an appropriate body?	X	☐	☐	☐
10. Do the conclusions drawn in the research report flow from the analysis, or interpretation, of the data?	X	☐	☐	☐

Overall appraisal: Include X Exclude ☐ Seek further info ☐

Comments [Including reason for exclusion]

3] JBI CRITICAL APPRAISAL CHECKLIST FOR QUALITATIVE RESEARCH

Reviewer S.Magowan

Date 17/05/22

Author Nahal MS, Axelsson AB, Imam A, Wigert H. Palestinian children's narratives about living with spina bifida: Stigma, vulnerability, and social exclusion. Child: care, health and development. 2019;45[1]:54-62.

	Yes	No	Unclear	Not applicable
1. Is there congruity between the stated philosophical perspective and the research methodology?	☐	☐	X	☐
2. Is there congruity between the research methodology and the research question or objectives?	X	☐	☐	☐
3. Is there congruity between the research methodology and the methods used to collect data?	X	☐	☐	☐
4. Is there congruity between the research methodology and the representation and analysis of data?	X	☐	☐	☐
5. Is there congruity between the research methodology and the interpretation of results?	X	☐	☐	☐
6. Is there a statement locating the researcher culturally or theoretically?	☐	X	☐	☐
7. Is the influence of the researcher on the research, and vice- versa, addressed?	☐	X	☐	☐
8. Are participants, and their voices, adequately represented?	X	☐	☐	☐
9. Is the research ethical according to current criteria or, for recent studies, and is there evidence of ethical approval by an appropriate body?	X	☐	☐	☐
10. Do the conclusions drawn in the research report flow from the analysis, or interpretation, of the data?	X	☐	☐	☐

Overall appraisal: Include X Exclude ☐ Seek further info ☐

Comments [Including reason for exclusion]

4] JBI CRITICAL APPRAISAL CHECKLIST FOR QUALITATIVE RESEARCH

Reviewer S.Magowan
Date 17/05/22

Author Page DT, Coetzee BJ. South African adolescents living with spina bifida: contributors and hindrances to well-being. Disability and rehabilitation. 2021;43[7]:920-8.

	Yes	No	Unclear	Not applicable
1. Is there congruity between the stated philosophical perspective and the research methodology?	☐	☐	X	☐
2. Is there congruity between the research methodology and the research question or objectives?	X	☐	☐	☐
3. Is there congruity between the research methodology and the methods used to collect data?	X	☐	☐	☐
4. Is there congruity between the research methodology and the representation and analysis of data?	X	☐	☐	☐
5. Is there congruity between the research methodology and the interpretation of results?	X	☐	☐	☐
6. Is there a statement locating the researcher culturally or theoretically?	☐	X	☐	☐
7. Is the influence of the researcher on the research, and vice- versa, addressed?	☐	X	☐	☐
8. Are participants, and their voices, adequately represented?	X	☐	☐	☐
9. Is the research ethical according to current criteria or, for recent studies, and is there evidence of ethical approval by an appropriate body?	X	☐	☐	☐
10. Do the conclusions drawn in the research report flow from the analysis, or interpretation, of the data?	X	☐	☐	☐

Overall appraisal: Include X Exclude ☐ Seek further info ☐

Comments [Including reason for exclusion]

5] JBI CRITICAL APPRAISAL CHECKLIST FOR QUALITATIVE RESEARCH

Reviewer S.Magowan

Date 17/05/22

Author Pfeiffer KM, Brod M, Smith A, Viuff D, Ota S, Charlton RW. Functioning and well-being in older children and adolescents with achondroplasia: A qualitative study. American Journal of Medical Genetics, Part A. 2021.

	Yes	No	Unclear	Not applicable
1. Is there congruity between the stated philosophical perspective and the research methodology?	X	☐	☐	☐
2. Is there congruity between the research methodology and the research question or objectives?	X	☐	☐	☐
3. Is there congruity between the research methodology and the methods used to collect data?	X	☐	☐	☐
4. Is there congruity between the research methodology and the representation and analysis of data?	X	☐	☐	☐
5. Is there congruity between the research methodology and the interpretation of results?	X	☐	☐	☐
6. Is there a statement locating the researcher culturally or theoretically?	☐	☐	X	☐
7. Is the influence of the researcher on the research, and vice- versa, addressed?	☐	☐	X	☐
8. Are participants, and their voices, adequately represented?	☐	☐	X	☐
9. Is the research ethical according to current criteria or, for recent studies, and is there evidence of ethical approval by an appropriate body?	X	☐	☐	☐
10. Do the conclusions drawn in the research report flow from the analysis, or interpretation, of the data?	X	☐	☐	☐

Overall appraisal: Include ☐ Exclude ☐ Seek further info X

Comments [Including reason for exclusion]

6] JBI CRITICAL APPRAISAL CHECKLIST FOR QUALITATIVE RESEARCH

Reviewer S.Magowan
Date 17/05/22

Author Volfson Z, McPherson AC, Tomasone JR, Faulkner GE, Arbour-Nicitopoulos KP. Examining factors of physical activity participation in youth with spina bifida using the Theoretical Domains Framework. Disability and Health Journal. 2020;13[4]:100922.

	Yes	No	Unclear	Not applicable
1. Is there congruity between the stated philosophical perspective and the research methodology?	X	☐	☐	☐
2. Is there congruity between the research methodology and the research question or objectives?	X	☐	☐	☐
3. Is there congruity between the research methodology and the methods used to collect data?	X	☐	☐	☐
4. Is there congruity between the research methodology and the representation and analysis of data?	X	☐	☐	☐
5. Is there congruity between the research methodology and the interpretation of results?	X	☐	☐	☐
6. Is there a statement locating the researcher culturally or theoretically?	X	☐	☐	☐
7. Is the influence of the researcher on the research, and vice- versa, addressed?	☐	☐	X	☐
8. Are participants, and their voices, adequately represented?	X	☐	☐	☐
9. Is the research ethical according to current criteria or, for recent studies, and is there evidence of ethical approval by an appropriate body?	X	☐	☐	☐
10. Do the conclusions drawn in the research report flow from the analysis, or interpretation, of the data?	X	☐	☐	☐

Overall appraisal: Include X Exclude ☐ Seek further info ☐

Comments [Including reason for exclusion]

7] JBI CRITICAL APPRAISAL CHECKLIST FOR QUALITATIVE RESEARCH

Reviewer S.Magowan
Date 17/05/22

Author Willson LR, Klootwyk M, Rogers LG, Shearer K, Southon S, Sasseville C. Timelines for returning to physical activity following pediatric spinal surgery: recommendations from the literature and preliminary data. BMC Research Notes. 2021;14:1-7.

	Yes	No	Unclear	Not applicable
1. Is there congruity between the stated philosophical perspective and the research methodology?	☐	☐	X	☐
2. Is there congruity between the research methodology and the research question or objectives?	☐	☐	X	☐
3. Is there congruity between the research methodology and the methods used to collect data?	X	☐	☐	☐
4. Is there congruity between the research methodology and the representation and analysis of data?	X	☐	☐	☐
5. Is there congruity between the research methodology and the interpretation of results?	X	☐	☐	☐
6. Is there a statement locating the researcher culturally or theoretically?	☐	X	☐	☐
7. Is the influence of the researcher on the research, and vice- versa, addressed?	☐	X	☐	☐
8. Are participants, and their voices, adequately represented?	☐	☐	X	☐
9. Is the research ethical according to current criteria or, for recent studies, and is there evidence of ethical approval by an appropriate body?	X	☐	☐	☐
10. Do the conclusions drawn in the research report flow from the analysis, or interpretation, of the data?	X	☐	☐	☐

Overall appraisal: Include ☐ Exclude ☐ Seek further info X

Comments [Including reason for exclusion]

8] JBI CRITICAL APPRAISAL CHECKLIST FOR QUALITATIVE RESEARCH

Reviewer S.Magowan

Date 17/05/22

Author Fischer, N., Church, P., Lyons, J., & McPherson, A. C. [2015]. A qualitative exploration of the experiences of children with spina bifida and their parents around incontinence and social participation. Child: Care, Health and Development, 41[6], 954–962.

	Yes	No	Unclear	Not applicable
1. Is there congruity between the stated philosophical perspective and the research methodology?	☐	☐	X	☐
2. Is there congruity between the research methodology and the research question or objectives?	X	☐	☐	☐
3. Is there congruity between the research methodology and the methods used to collect data?	X	☐	☐	☐
4. Is there congruity between the research methodology and the representation and analysis of data?	X	☐	☐	☐
5. Is there congruity between the research methodology and the interpretation of results?	X	☐	☐	☐
6. Is there a statement locating the researcher culturally or theoretically?	X	☐	☐	☐
7. Is the influence of the researcher on the research, and vice- versa, addressed?	☐	☐	X	☐
8. Are participants, and their voices, adequately represented?	X	☐	☐	☐
9. Is the research ethical according to current criteria or, for recent studies, and is there evidence of ethical approval by an appropriate body?	X	☐	☐	☐
10. Do the conclusions drawn in the research report flow from the analysis, or interpretation, of the data?	X	☐	☐	☐

Overall appraisal: Include X Exclude ☐ Seek further info ☐

Comments [Including reason for exclusion]

9] JBI CRITICAL APPRAISAL CHECKLIST FOR QUALITATIVE RESEARCH

Reviewer S.Magowan

Date 17/05/22

Author Strömfors L, Wilhelmsson S, Falk L, et al. Experiences among children and adolescents of living with spina bifida and their visions of the future. Disabil Rehabil. 2017;39: 261–271

	Yes	No	Unclear	Not applicable
1. Is there congruity between the stated philosophical perspective and the research methodology?	☐	☐	X	☐
2. Is there congruity between the research methodology and the research question or objectives?	X	☐	☐	☐
3. Is there congruity between the research methodology and the methods used to collect data?	X	☐	☐	☐
4. Is there congruity between the research methodology and the representation and analysis of data?	X	☐	☐	☐
5. Is there congruity between the research methodology and the interpretation of results?	X	☐	☐	☐
6. Is there a statement locating the researcher culturally or theoretically?	☐	X	☐	☐
7. Is the influence of the researcher on the research, and vice- versa, addressed?	☐	X	☐	☐
8. Are participants, and their voices, adequately represented?	X	☐	☐	☐
9. Is the research ethical according to current criteria or, for recent studies, and is there evidence of ethical approval by an appropriate body?	X	☐	☐	☐
10. Do the conclusions drawn in the research report flow from the analysis, or interpretation, of the data?	X	☐	☐	☐

Overall appraisal: Include X Exclude ☐ Seek further info ☐

Comments [Including reason for exclusion]

<u>Supplementary File S2:</u> Participant Quotes

Reference	Quote	Parent or Child	Age of Child [years]	Gender	Condition
Bloemen MAT, Verschuren O, van Mechelen C, Borst HE, de Leeuw AJ, van der Hoef M, et al. Personal and environmental factors to consider when aiming to improve participation in physical activity in children with Spina Bifida: a qualitative study. BMC neurology. 2015;15:11. [68]	I always self-propel my wheelchair,....at a certain point,... you have to do it yourself later on	Child	8–18	Unknown	Spinal Bifida
	the complication is, that the stimulation always has to come from us....what I experience from my healthy children,... is they ask us for help,.... and say 'now you have to help me because ..... I want to do this and that', they ask. He doesn't ask, it always has to be stimulated by us	Parent	4–7	Unknown	Spinal Bifida
	if you play sports, you get energy....., you'll become fit and yes you'll notice	Child	8-18	Unknown	Spinal Bifida
	because you're around people, you make contact with people, sometimes you make friends	Child	8-18	Unknown	Spinal Bifida
	because I dare to do things now that others don't dare	Child	8-18	Female	Spinal Bifida
	Yes, I would also like to do that, for my self-confidence because...if you fall, you know what to do	Child	8-18	Female	Spinal Bifida
	really an obstacle...., every 3.5 hour it has to happen.... so you always have to plan ahead, or you have to go back and forth...., you always have to say 'it's not possible to come directly after school because he has to go to the toilet first', it is even a bigger obstacle than the handicap, you always have to be there as a parent...	Parent	8-18	Male	Spinal Bifida
	Wheelchair training, that is very important I think, ...that they really learn to go up and down stairs..... ....she can do much more now....a lot of places are not adjusted for wheel-chairs ....and you can just go....your life becomes a lot more fun	Parent	8-18	Female	Spinal Bifida
	being unfit" because "you get tired more easily	Child	8-18	Unknown	Spinal Bifida
	I think partly maybe the way I was brought up, because my parents they always say, you have to propel yourself as much as possible, because your fitness will increase......if I start to complain 'I'm tired, I want to go home', well, they ignore it......I think it is ok, I think I will benefit later on. On the other hand, at that moment when I'm tired and they don't want to	Child	8-18	Unknown	Spinal Bifida

	push me, I am mad [laughing]				
	sometimes I see handicapped children....older than I am, and they are treated like they are much younger and then I think, you just can't do that	Child	8-18	Female	Spinal Bifida
	well, I think it is like that, because they usually think that you're also mentally handicapped and that's why they think oh, he's not that smart	Child	8-18	Male	Spinal Bifida
	…they easily think that activities are too hard....if I for example say 'I want to do this and that' he will say 'that is too hard for you.....what if something happens...'....well. It is difficult to say otherwise...	Child	8-18	Female	Spinal Bifida
	[P] We now have a teacher who absolutely doesn't want to make adjustments in the physical education class. They just say 'if he can't do it, he can't do it'. We had a huge discussion about his grade for physical education this year. She didn't want to give a higher grade than a C, because, well, that was just not possible.	Parent	8-18	Male	Spinal Bifida
	It has to be in the neighborhood....so you can go by yourself...so your parents don't have to take you. Yes, because when you grow older, it is annoying always having your parents around	Child	8-18	Unknown	Spinal Bifida
	there are a lot of enthusiastic people who said it really fits in our club, and we're going to take care of it!	Parent	4-7	Unknown	Spinal Bifida
	[P] they have a huge role and they think it is important	Parent	8-18	Unknown	Spinal Bifida
	[P] you notice that they're working on it, but I think it should go faster	Parent	8-18	Unknown	Spinal Bifida
	you can achieve the same things with an assistive device as an able-bodied person..., a wheelchair is a replacement of your legs.....but then you need good equipment... I should not have to adjust to my equipment...it should exactly be the other way around	Child	8-18	Unknown	Spinal Bifida
	Since September, she can't handbike anymore, ...if everything is ok again, the summer is probably over, that's such a waste,...her friend lives 3 kilometer away, she can easily bike it, but now we have to take her,....she is	Parent	8-18	Female	Spinal Bifida

	very limited because of assistive mobility devices that do not work				
	A lot of things you have to find out yourself...I do miss that.....I think, if you're in a hospital, we visit the hospital regularly, that there should be.....more information...and listening what the child wants and I do miss that......they ask for example 'how is it', 'yes everything goes well' he [the child] says, well he always says everything goes well......but I think....you should ask 'what else do you want, how is it going with playing sports, do you play sports', it is always about what school do you go to and that's that	Parent	8-18	Male	Spinal Bifida
	especially one on one, somebody who says.....that's all available, what kind of child do you have, what kind of situation, where do you live, what are you looking for, leading to something concrete	Parent	4-7	Unknown	Spinal Bifida
	In third grade they spent a lesson on him, they have this book, ...it is about a boy with SB....the teacher read it aloud and then they talked about it	Parent	8-18	Male	Spinal Bifida
	we always had support from a regional expertise centre and a therapist comes in ones every so many times	Parent	8-18	Unknown	Spinal Bifida
Gorzkowski J, Kelly EH, Klaas SJ, Vogel LC. Obstacles to community participation among youth with spinal cord injury. The journal of spinal cord medicine. 2011;34[6]:576-85. [69]	not all areas are wheelchair accessible	Caregiver	Unknown	Unknown	Spinal Cord Injury
	lack of knowledge in the community	Caregiver	Unknown	Unknown	Spinal Cord Injury
	there are not enough local community activities that can include someone with a physical disability ... most programming is geared towards individuals with cognitive disabilities	Caregiver	Unknown	Unknown	Spinal Cord Injury
	bowel program	Caregiver	Unknown	Unknown	Spinal Cord Injury
	level of injury too high	Caregiver	Unknown	Unknown	Spinal Cord Injury
	just the problems associated with being in a wheelchair. Sometimes she will just stay at home because she doesn't want the hassle of loading the chair, unloading the chair, etc.	Caregiver	Unknown	Unknown	Spinal Cord Injury
	family size [four kids] causes practical concerns	Caregiver	Unknown	Unknown	Spinal Cord Injury
	timing and scheduling. Mak[ing] my schedule fit with his schedule	Caregiver	Unknown	Unknown	Spinal Cord Injury
	the thing that limits our child's activities is that there are not any	Caregiver	Unknown	Unknown	Spinal Cord Injury

	carpool options – so we have to ensure that my wife or I are available for transportation				
	self esteem	Caregiver	Unknown	Unknown	Spinal Cord Injury
	... my child’s lack of desire to participate. My child is somewhat shy and not particularly outgoing. This was also true prior to his injury	Caregiver	Unknown	Unknown	Spinal Cord Injury
	feeling that he ‘cannot do that	Caregiver	Unknown	Unknown	Spinal Cord Injury
	would not fit in	Caregiver	Unknown	Unknown	Spinal Cord Injury
	he has friends but I do not feel that the kids would encourage my child to play on their sports team; they would feel that it may make them lose	Caregiver	Unknown	Unknown	Spinal Cord Injury
	people worry that he will get hurt and they will be liable	Caregiver	Unknown	Unknown	Spinal Cord Injury
	PE [physical education] teacher in high school did not think she could partici- pate in PE although she had been doing PE since fifth grade, so they put her in marching band	Caregiver	Unknown	Unknown	Spinal Cord Injury
	weather [winter bad roads]	Caregiver	Unknown	Unknown	Spinal Cord Injury
	too young	Caregiver	Unknown	Unknown	Spinal Cord Injury
	the fear of getting more injuries	Caregiver	Unknown	Unknown	Spinal Cord Injury
Nahal MS, Axelsson AB, Imam A, Wigert H. Palestinian children's narratives about living with spina bifida: Stigma, vulnerability, and social exclusion. Child: care, health and development. 2019;45[1]:54-62.	I hate the wheelchair ... I hope to get rid of it ... It annoys me. I cannot be like others ... I often think ‘Why me? Why am I not a normal child like others?	Child	14 years	Male	Spinal Bifida
	“Everything is difficult in my life. I feel tired of living with incontinence and diapers ... I hate the catheterization ... I wondered why children like me with Spinal bifida should stay alive.”	Child	12 years	Female	Spinal Bifida
	I pretend to use the bathroom while I am at school ... So that other children think I'm normal and can use the bathroom as they do.	Child	10 years	Male	Spinal Bifida
	The school took me out of my comfort zone ... I became aware that I'm different from some children who stared at me and made fun of my shaky walk.	Child	12 years	Female	Spinal Bifida
	My brothers are similar to me. The only difference is that they can walk. But I can crawl and control the wheelchair.	Child	9 years	Male	Spinal Bifida
	I was excluded from all the outings, such as field trips and physical education classes ... I like to play football. But I couldn't participate in	Child	14 years	Male	Spinal Bifida

	that ... The teachers said ‘It is difficult. You just sit there and watch the other children.				
	I hate school ... I feel that many children give me looks and put me down ... because they discovered my incontinence ... They always make me feel afraid and ashamed ... I usually like to stay alone.	Child	10 years	Male	Spinal Bifida
	I prefer to stay home ... You know how people in our society deal with such cases ... they look at me curiously ... I do not like to hear words of sympathy ... It's really hard ... I'm a human. And I want people to respect me.	Child	16 years	Male	Spinal Bifida
	I would like to join a public sports club. I love swimming and playing football with other children ... I am very sad ... It is difficult to reach these areas ... I am not allowed to participate ... I prefer to stay home.	Child	16 years	Male	Spinal Bifida
	Paralysis makes my situation tragic ... I was just sitting near the heater. I did not notice that my legs were burnt until my mother entered the room and started shouting.	Child	14 years	Male	Spinal Bifida
	Three years ago, I felt depressed. I tried to end my life ... Then my parents talked to me and helped me continue this miserable life.	Child	12 years	Female	Spinal Bifida
	My mother taught me that I am a gift from God. She told me I will walk in heaven and will gain later whatever I have lost in this life.	Child	7 years	Female	Spinal Bifida
	The religious beliefs I learned from my family and from my mum still help me. My parents took me to Saudi Arabia to request prayers ... to protect me and my family.	Child	16 years	Male	Spinal Bifida
	My friends, mainly cousins, are very helpful. I am happy to have them in my life ... They often encourage me. I'm not allowed to stay out with them for a long period ... but I'm lucky that I can keep contact with them through the computer.	Child	15 years	Male	Spinal Bifida
Page DT, Coetzee BJ. South African adolescents living with spina bifida: contributors and hindrances to well-	I told him I’m not alone here your dad is also there you must go to him, but he doesn’t want to	Caregiver	53 Years	Female	Spinal Bifida
	And I can only speak of my experience, and say that I was blessed in many ways, with a big family	Caregiver	42 Years	Female	Spinal Bifida

being. Disability and rehabilitation. 2021;43[7]:920-8.	support				
	Yes just they mustn't be shy for their child ... The people must adjust around them, and the families must try to adapt to how they are man. They mustn't feel out because of their disability	Caregiver	45 Years	Female	Spinal Bifida
	I think the only good influences that she has are the ones that are in the neighbourhood, because ... I don't know they not negative ...	Caregiver	43 Years	Male	Spinal Bifida
	And what I really admire at school, is that the children, I mean they help each other, they care for each other, which at mainstream schools they don't do that!	Caregiver	45 Years	Female	Spinal Bifida
	I find it to be one of the best schools. So, at school they are also motivated because they see a lot of them around and also they see some of the ex-students who come and motivate...	Caregiver	43 Years	Male	Spinal Bifida
	...And you know, they would like, know he can't kick the ball, so they'll throw the ball to each other, and then they play hide and seek or you know, those type of things ...	Caregiver	42 Years	Female	Spinal Bifida
	[adolescent participant] at school was involved in the wheelchair dance competitions and then there's... volleyball... That she played at school. They put them in a lot of stuff at school. She's participating in all stuff that we go watch	Caregiver	Unknown	Female	Spinal Bifida
	Tell them to join sports, that's all I can tell. I think, first I didn't want to do athletics, now I can beat everyone.	Child	15 years	Male	Spinal Bifida
	We have done so many things, trying to inculcate in her she needs to do what she's supposed to be doing all the time. She needs to be consistent	Caregiver	43 Years	Male	Spinal Bifida
	I always encourage him and I always tell him that things are gonna get better. Things will get better and he has big dreams, you know, he always say he wants to drive ...	Caregiver	45 Years	Female	Spinal Bifida
	What gives my life meaning is also helping and being there for elderly people and helping those that need my help...	Child	13 years	Female	Spinal Bifida
	God had chosen them as a special child	Caregiver	Unknown	Female	Spinal Bifida
	I'm grateful that I've got family, great friends...I'm grateful because God	Child	13 years	Female	Spinal Bifida

	created me, I'm unique. Thankful for the love of my family, what they gave ...				
	There's no support... So we're trying to do that, like maybe on one occasion we have like maybe the, the session for the boys, where they'll do shaving, or you know, self-care"	Caregiver	42 Years	Female	Spinal Bifida
	"Yes, I do think that if it's put out there and the media is aware of it that it would change things. Because I find that people tend to hide their kids	Caregiver	42 Years	Female	Spinal Bifida
	I'm just worried about her health because ... she's don't know, looks like [adolescent participant] don't see the importance of her stuff that she must do	Caregiver	Unknown	Female	Spinal Bifida
	Cause I don't wanna be like in a wheelchair. It's not fun to be in. Then I can do nothing, like say my now I wanna go play soccer then I can't cause I'm here in. And then I can't do what other children can do	Child	13 years	Male	Spinal Bifida
	You know ... And that is why I always tell him, the wheelchair doesn't define you. You are your own person, that is just your means to get around... But it doesn't define you as a person because you are your own person. Whatever choices you make, then that's the choices you're gonna live with	Caregiver	42 years	Female	Spinal Bifida
	I don't know, [adolescent participant]...if she gets um supported by her friends at school because she was she was usually the child that was bullied at school. Because I think it was two years ago, not last year, but she came home with her eye with a blue eye that one of the children did hit her. So I'm not sure	Caregiver	Unknown	Female	Spinal Bifida
	I...my mommy don't know so... I smoke, but my mommy knows I drink a little	Child	13 years	Female	Spinal Bifida
	You know, so a lot of people know nothing about spina bifida. A lot of people even our families they have no idea. And even when they see children who look like ... they have no idea and yet there are so many people even in our country living with spina bifida	Caregiver	43 years	Male	Spinal Bifida
	Of those killing, killing kids, girls, taking girls and grabbing them and	Child	13 years	Female	Spinal Bifida

	killing taking the body parts. I don't go outside anymore. I never go outside				
	We don't go out like to the mall, we just see each other at school and on the streets	Child	13 years	Female	Spinal Bifida
	No, I didn't take him yet, must walk there and all the skollies [criminals], gangsters is that side	Caregiver	53 years	Female	Spinal Bifida
	When asked "when do you feel most happy": That would be only like half the time, or like at school. Like ja	Child	16 years	Male	Spinal Bifida
	He's not happy at the moment. I don't know when he will accept it. But he is not happy at all	Caregiver	75 years	Male	Spinal Bifida
Pfeiffer KM, Brod M, Smith A, Viuff D, Ota S, Charlton RW. Functioning and well-being in older children and adolescents with achondroplasia: A qualitative study. American Journal of Medical Genetics, Part A. 2021.	Pain; Back pain, Joint pain, Leg pain	29 Children	9-12yrs = 12<br>12-15yrs = 8<br>15-18yrs = 9	Unknown	Achondroplasia
	Low stamina/tiring easily	26 Children	9-12yrs = 9<br>12-15yrs = 8<br>15-18yrs = 9	Unknown	Achondroplasia
	Teeth crowding / misalignment	22 Children	9-12yrs = 7<br>12-15yrs = 8<br>15-18yrs = 7	Unknown	Achondroplasia
	Ear infections / fluid in the ear	11 Children	9-12yrs = 5<br>12-15yrs = 3<br>15-18yrs = 3	Unknown	Achondroplasia
	Hearing problems / loss	11 Children	9-12yrs = 5<br>12-15yrs = 2<br>15-18yrs = 4	Unknown	Achondroplasia
	Overweight / obesity	9 Children	9-12yrs = 0<br>12-15yrs = 5<br>15-18yrs = 4	Unknown	Achondroplasia
	Use of adaptive devices [E.g. step stools]	32 Children	9-12yrs = 13<br>12-15yrs = 9<br>15-18yrs = 10	Unknown	Achondroplasia
	Difficulty reaching objects / high places	27 Children	9-12yrs = 11<br>12-15yrs = 8<br>15-18yrs = 8	Unknown	Achondroplasia
	Need assistance from others for tasks	26 Children	9-12yrs = 9<br>12-15yrs = 8<br>15-18yrs = 9	Unknown	Achondroplasia
	Difficulty walking long distances	24 Children	9-12yrs = 8<br>12-15yrs = 7<br>15-18yrs = 9	Unknown	Achondroplasia
	Issues with prolonged sitting or sitting without support	18 Children	9-12yrs = 9<br>12-15yrs = 4<br>15-18yrs = 5	Unknown	Achondroplasia
	Difficulty being physically active	15 Children	9-12yrs = 4<br>12-15yrs = 5<br>15-18yrs = 6	Unknown	Achondroplasia
	Difficulty running	14 Children	9-12yrs = 10	Unknown	Achondroplasia

			12-15yrs = 4<br>15-18yrs = 0		
	Issues bathing / washing / grooming self	8 Children	9-12yrs = 5<br>12-15yrs = 2<br>15-18yrs = 1	Unknown	Achondroplasia
	Difficulty with stairs or steps	7 Children	9-12yrs = 1<br>12-15yrs = 2<br>15-18yrs = 4	Unknown	Achondroplasia
	Communication issues [e.g. due to trouble hearing]	7 Children	9-12yrs = 2<br>12-15yrs = 2<br>15-18yrs = 3	Unknown	Achondroplasia
	Difficulty lifting / carrying objects	7 Children	9-12yrs = 2<br>12-15yrs = 3<br>15-18yrs = 2	Unknown	Achondroplasia
	Challenges associated with travel	7 Children	9-12yrs = 1<br>12-15yrs = 2<br>15-18yrs = 4	Unknown	Achondroplasia
	Difficulty hiking / climbing	7 Children	9-12yrs = 3<br>12-15yrs = 2<br>15-18yrs = 2	Unknown	Achondroplasia
	Feeling different	20 Children	9-12yrs = 8<br>12-15yrs = 7<br>15-18yrs = 5	Unknown	Achondroplasia
	Worried / scared	15 Children	9-12yrs = 5<br>12-15yrs = 5<br>15-18yrs = 5	Unknown	Achondroplasia
	Embarrassed / self-conscious	15 Children	9-12yrs = 4<br>12-15yrs = 7<br>15-18yrs = 4	Unknown	Achondroplasia
	Frustrated / annoyed	13 Children	9-12yrs = 4<br>12-15yrs = 3<br>15-18yrs = 6	Unknown	Achondroplasia
	Sad / hurt	13 Children	9-12yrs = 7<br>12-15yrs = 3<br>15-18yrs = 3	Unknown	Achondroplasia
	Feel happy / experience joy	10 Children	9-12yrs = 2<br>12-15yrs = 3<br>15-18yrs = 5	Unknown	Achondroplasia
	Sense that life is difficult / challenging	8 Children	9-12yrs = 1<br>12-15yrs = 3<br>15-18yrs = 4	Unknown	Achondroplasia
	Angry / mad	7 Children	9-12yrs = 3<br>12-15yrs = 3<br>15-18yrs = 1	Unknown	Achondroplasia
	Bothered	7 Children	9-12yrs = 1<br>12-15yrs = 5<br>15-18yrs = 1	Unknown	Achondroplasia
	Difficulty participating in sports / physical play	26 Children	9-12yrs = 10<br>12-15yrs = 8<br>15-18yrs = 8	Unknown	Achondroplasia
	Needing to explain achondroplasia to others	26 Children	9-12yrs = 12<br>12-15yrs = 6	Unknown	Achondroplasia

			15-18yrs = 8		
	Being treated as younger than age	24 Children	9-12yrs = 11<br>12-15yrs = 5<br>15-18yrs = 8	Unknown	Achondroplasia
	Issues participating in social activities	23 Children	9-12yrs = 9<br>12-15yrs = 8<br>15-18yrs = 6	Unknown	Achondroplasia
	Teasing / bullying	21 Children	9-12yrs = 11<br>12-15yrs = 5<br>15-18yrs = 5	Unknown	Achondroplasia
	Negative attention in public [e.g. staring, pointing]	20 Children	9-12yrs = 7<br>12-15yrs = 7<br>15-18yrs = 6	Unknown	Achondroplasia
	Friendships / social activities through a community of people with dwarfism	16 Children	9-12yrs = 5<br>12-15yrs = 5<br>15-18yrs = 6	Unknown	Achondroplasia
	Difficulty keeping up with other children their age physically	14 Children	9-12yrs = 8<br>12-15yrs = 3<br>15-18yrs = 3	Unknown	Achondroplasia
	Being stigmatised	12 Children	9-12yrs = 3<br>12-15yrs = 6<br>15-18yrs = 3	Unknown	Achondroplasia
	Peers treat differently	10 Children	9-12yrs = 5<br>12-15yrs = 0<br>15-18yrs = 5	Unknown	Achondroplasia
	Positive impacts on friendships	9 Children	9-12yrs = 3<br>12-15yrs = 5<br>15-18yrs = 1	Unknown	Achondroplasia
	Special adaptations / accommodations at school	28 Children	9-12yrs = 12<br>12-15yrs = 7<br>15-18yrs = 9	Unknown	Achondroplasia
	Difficulty participating in physical education [P.E]	26 Children	9-12yrs = 12<br>12-15yrs = 7<br>15-18yrs = 7	Unknown	Achondroplasia
	Issues participating in class / schoolwork	19 Children	9-12yrs = 9<br>12-15yrs = 3<br>15-18yrs = 7	Unknown	Achondroplasia
	Missed school days / time	17 Children	9-12yrs = 4<br>12-15yrs = 5<br>15-18yrs = 8	Unknown	Achondroplasia
	Issues participating in school activities / field trips	13 Children	9-12yrs = 4<br>12-15yrs = 5<br>15-18yrs = 4	Unknown	Achondroplasia
	Difficulty getting from place to place at school	13 Children	9-12yrs = 3<br>12-15yrs = 2<br>15-18yrs = 8	Unknown	Achondroplasia
Volfson Z, McPherson AC, Tomasone JR, Faulkner GE, Arbour-Nicitopoulos KP. Examining factors of physical activity	[my coaches] have been super supportive and understanding. They adjust accordingly, and if I have to leave the ice they let the ref know and the game know. They accommodate and adjust.	Child	15yrs	Female	Spinal Bifida

participation in youth with spina bifida using the Theoretical Domains Framework. Disability and Health Journal. 2020;13[4]:100922.	fun factor	Multiple children	Unknown	Unknown	Spinal Bifida
	Our team is just a lot of fun, on ice and in the dressing room.	Child	16yrs	Male	Spinal Bifida
	I go to the gym at school … I usually work on the punching bag, weight stuff like that. I did take fitness course and I took gym for a while. As well as I saw my friends doing it and I guess I started to imitate them.	Child	17yrs	Male	Spinal Bifida
	[PA duration] mmm … around an hour I think … maybe a little more.	Child	17yrs	Male	Spinal Bifida
	Well my mom just signed me up for para swimming … I didn't want to, my mom forced me.	Child	16yrs	Female	Spinal Bifida
	Well I had a really great teacher so she would always include me in everything. She modified things when I got tired. I remember one time I got to play ping pong with people instead.	Child	16yrs	Female	Spinal Bifida
	… but still being aware if there is back pain … how much of it. Do I need to talk to my coach to see if they need to put someone on. I take some Tylenol if it gets really bad … I have a heating pad that I will lay on or we'll put muscle rub on my back before some games.	Child	17yrs	Female	Spinal Bifida
Willson LR, Klootwyk M, Rogers LG, Shearer K, Southon S, Sasseville C. Timelines for returning to physical activity following pediatric spinal surgery: recommendations from the literature and preliminary data. BMC Research Notes. 2021;14:1-7.	I talk to my mom and my dad but then also, if I'm doing something and its hurting or whatever I just stop. Or if I'm not really too sure about something, I'll do a little bit... or lift a portion of it if that's possible, and then if I'm okay I just kind of keep going and then if it hurts, I stop immediately	Child	Unknown	Unknown	Post-operative AIS surgery
	I'm really scared about getting hurt	Child	Unknown	Unknown	Post-operative AIS surgery
	It sucked for a while; I was a pretty active person so the first 6–7 months kind of sucked	Child	Unknown	Unknown	Post-operative AIS surgery
	I used to work out at lunch with my friends and I can't do that anymore...it's just sucky	Child	Unknown	Unknown	Post-operative AIS surgery
Fischer, N., Church, P., Lyons, J., & McPherson, A. C. [2015]. A qualitative exploration of the experiences of children with spina bifida and their parents around incontinence and social	[He's] just a regular, everyday kid	Parent	Male	10 years	Spinal Bifida
	She participates at school like any other child and participates in extracurricular activities like any other child and is treated like anyone else	Parent	Female	10 years	Spinal Bifida
	But we always have pushed him to be like other kids, to try to be as much like other kids	Parent	Male	16 years	Spinal Bifida

participation. Child: Care, Health and Development, 41[6], 954–962.	Sometimes it gets into my head and then I start thinking that I'm different and that – like I start believing them	Child	Female	10 years	Spinal Bifida
	I just don't want to be judged because I'm different	Child	Male	16 years	Spinal Bifida
	I went through very many emotional roller coasters to finally realise that I was not like everyone else and I had to accept that	Child	Female	14 years	Spinal Bifida
	I want her to feel like a big girl and be as much like the rest of them – her friends – as she can	Parent	Female	8 years	Spinal Bifida
	I don't want to be the same like everyone else. The only thing I want to be the same like everyone else is to wear panties	Child	Female	10 years	Spinal Bifida
	It's like this is my security, I guess...I might try it [wearing regular underwear]; I always keep saying I might try it soon but we'll see	Child	Female	18 years	Spinal Bifida
	I knew that I had to because my parents aren't going to be there in Grade 1 and I just didn't want everybody knowing and I didn't want everybody to be involved in it. I'm just like okay, I'm gonna do it.	Child	Female	9 years	Spinal Bifida
	Well, she's been doing [clean intermittent catheterization] since she was six, so she just decided to give it a try and get the mirror out....All it took was putting the idea in Caroline's head and she wanted to try it and well, within a couple of weeks she was doing it all by herself.	Parent	Female	8 years	Spinal Bifida
	It was a goal we were working towards....make her independent to make her take ownership of her situation, and if you do, this is what you could have, you get...a very small absorbent pad versus a pull up. So it worked for a little while and then she fell off the wagon.	Parent	Female	10 years	Spinal Bifida
	If he wants to go to a sledge hockey camp overnight and this kind of thing for a week, that's the kind of thing you need to have more independence with	Parent	Male	16 years	Spinal Bifida
	I was scared of missing something because I was the type of person that was like I want to be here and there and there, and I'm not thinking about going to the bathroom; that didn't like occur to me sometimes. I don't know why. I guess I was a stupid little kid	Child	Female	18 years	Spinal Bifida

	weekly sleepovers with friends	Child	Female	9 years	Spinal Bifida
	a consistent group of close friends that she spent time with every weekend	Child	Female	18 years	Spinal Bifida
	accepted by their peers, regardless of their disability	Parent	Female	8 & 9 years	Spinal Bifida
	So I just decided to tell them before they found out and got shocked and ran away screaming	Child	Female	9 years	Spinal Bifida
	people know about my disability but I make them think that it's only my legs that are part of it	Child	Male	17 years	Spinal Bifida
	If I go in there and there are girls in the bathroom I'll walk out just so they don't see my catheter	Child	Female	18 years	Spinal Bifida
	They make fun of me and they say like, "Oh, you stink"	Child	Female	10 years	Spinal Bifida
	played mainly with his younger brother's friends	Child	Male	10 years	Spinal Bifida
	daughter's friends were her cousins and kids from the neighbourhood who wanted to hang out at her house even when Talia was not home	Parent	Female	10 years	Spinal Bifida
	It doesn't matter if I'm hanging out with friends or if I'm by myself, I don't care either way. I'm sort of always happy so it doesn't really matter to me	Child	Male	17 years	Spinal Bifida
	People they just kind of like, they look at me and they are like...okay...the chair is all they see	Child	Female	14 years	Spinal Bifida
	Some of the friends I have [on Facebook] they'll send me a friend request and I'll say yes, but...when I go to school it's like they don't even know I exist or they don't even talk to me	Child	Female	10 years	Spinal Bifida
	Normally we have friends to our house for her, because a lot of people's homes are not accessible	Parent	Female	14 years	Spinal Bifida
	He plays sledge [hockey]. And he does wheelchair racing. He tried basketball. He's been in a kayak. He's done swimming....And yet the friends aren't there. I think it's more of a bowel-bladder issue	Parent	Male	16 years	Spinal Bifida
	Because if they see what's in the garbage, then they'll ask me questions. I don't really want to explain it to them	Child	Male	16 years	Spinal Bifida
	Whenever I run [my bladder] just lets go	Child	Female	10 years	Spinal Bifida
Strömfors L,	I think kind of positively, it [SB] is not	Child	Male	15 years	Spinal Bifida

Wilhelmsson S, Falk L, et al. Experiences among children and adolescents of living with spina bifida and their visions of the future. Disabil Rehabil. 2017;39: 261–271	a deadly disease and lots of people are worse off. Plus, most of the people with SB have problems walking, which I do not have				
	The reason that I have not told my friends is that I see how they are now. Young people, and now I mean people my age, they are kind of, what can I say, immature or they say certain things. They think that if a person is different, then that person is a freak or something like that. That is why I have not dared [to tell them about having SB]	Child	Male	15 years	Spinal Bifida
	[laughter] I am quite good at exercising even though that I am not able to walk or run far, but I ride my bicycle. I bike a lot and kind of [pause] walk anyway [laughter]	Child	Female	12 years	Spinal Bifida
	those [persons with SB] that I have met have had a lot more problems than me, with difficulties in walking at all [not audible], so I think I am one of those that are less affected	Child	Female	12 years	Spinal Bifida
	Well, I can join them. I can move pretty fast, although not as fast [as the peers]	Child	Female	10 years	Spinal Bifida
	I probably do not get my homework done as fast as them [peers without SB] and I probably have more difficulties remembering things. And then there is the fact that you have to sneak out sometimes to go to the toilet	Child	Male	17 years	Spinal Bifida
	It [using the toilet] does not take more than five or six minutes, so I doubt that I miss very much [of lecture time]	Child	Male	17 years	Spinal Bifida
	But sometimes maybe it. . . Sometimes I cannot bother to do it, and then I throw them [the catheters] away	Child	Female	11 years	Spinal Bifida
	...but when I was in pre-school the assistant even helped me with peeing and that kind of thing. But then when I started school I had to learn to do it myself, and then the assistant only gave support [with planning schoolwork for example]	Child	Female	14 years	Spinal Bifida
	There are not a lot of things that can worry me. It is like, I improvise. If anything comes up that I cannot solve, we [participant and parents] will improvise. Then we will solve it when the problem arises	Child	Male	17 years	Spinal Bifida

	The thing is that I am not good at mathematics and I have noticed that you should be [to manage the desired occupation] [laughter]. I have some difficulties with mathematics, and then [. . .] I have difficulties remembering things, like I said, but I will probably have to. . . I have to practice so that I can remember [...] Writing things down, I have to pull myself together on that [laughter]. It is this kind of thing that I might worry a bit about that it will not work out	Child	Female	14 years	Spinal Bifida
	Maybe not completely, but as good as. . .there is always something that you maybe will see as a problem later in life. I do not really know what now, actually	Child	Male	17 years	Spinal Bifida
	Well, I have to do a bit of checking of the various ways you can do it [manage the toilet] and then practice the one [way of managing the toilet] that seems best, and it should be fine [P7, 17-year-old boy in a wheelchair].	Child	Male	17 years	Spinal Bifida

Supplementary File S3: Audit Trail

<table>
<tr>
<th>Theme [from column 1]</th>
<th>Sub-theme [end column currently]</th>
<th>Definition and Quotes

Definition of subtheme:

Uniqueness from conditions:
Congenital: x papers identified …

Quotes from papers:</th>
<th>Number of studies supporting</th>
</tr>
<tr>
<td>Biological Factors</td>
<td>Biological factors apparent. Identifying the range of challenges.

Focus on meaning and impact of biological factor on physical activity.</td>
<td><u>Definition:</u> What physical movement causes in terms of pain or gauging what level is appropriate.

<u>Uniqueness:</u>

<table>
<tr><th></th><th>Proportions [Quotes = 9]</th></tr>
<tr><td>Age of child / adolescent</td><td>1x 8-18yrs,
1x 17yrs,
2x 16yrs [same individual]
1x 15yrs
1x 14yrs
2x 12yrs
1x10yrs</td></tr>
<tr><td>Gender of child / adolescent</td><td>Male = 3,
Female = 6</td></tr>
<tr><td>Condition</td><td>SB = 9</td></tr>
<tr><td>Parent /Child</td><td>Parent =3 ,
Child / Adolescent = 6</td></tr>
</table>

<u>Quotes:</u>

"'....they easily think that activities are too hard....if I for example say 'I want to do this and that' he will say 'that is too hard for you.....what if something happens...'." [1] Female, Child age 8-18yrs, SB</td>
<td>1, 6, 8, 9</td>
</tr>
</table>

'… but still being aware if there is back pain … how much of it' [Dina] Female, 17yrs, Caucasian, no mobility aid, SB [6]

One father mentioned that he pushed his son to walk instead of using a wheelchair, even though this often resulted in blisters because of the lack of sensation in his feet: 'But we always have pushed him to be like other kids, to try to be as much like other kids'. [father of Landon, 16 years old]. Parent, Landon Male, 16yrs, grade 10, walks with braces, SB L4 myelomeningocele [8]

Additionally, it was viewed as a barrier to participation when children were unable to manage their incontinence; Landon's father mentioned, 'If he wants to go to a sledge hockey camp overnight and this kind of thing for a week, that's the kind of thing you need to have more independence with' [8]. Parent, Landon Male, 16yrs, grade 10, walks with braces, SB L4 myelomeningocele

For some children, mobility concerns limited their ability to engage in certain peer interactions, as Jasmin's mom discussed: 'Normally we have friends to our house for her, because a lot of people's homes are not accessible.' [8]. Parent, Jasmin, female, 14yrs, uses wheelchair, SB L4 lipomyelomeningocele

Positive attitudes could also arise from a sense of gratitude for having a relatively mild condition:
I think kind of positively, it [SB] is not a deadly disease and lots of people are worse off. Plus, most of the people with SB have problems walking, which I do not have [P6, 15-year-old boy walking] SB [9].

'[laughter] I am quite good at exercising even though that I am not able to walk or run far, but I ride my bicycle. I bike a lot and kind of [pause] walk anyway [laughter]' [P3, 12-year-old girl walking] SB [9].

Well, I can join them. I can move pretty fast, although not as fast [as the peers] [P1, 10-year-old girl using ambulatory devices/wheelchair] SB. [9].

those [persons with SB] that I have met have had a lot more problems than me, with difficulties in walking at all [not audible], so I think I am one of those that are less affected [P3, 12-year-old girl walking] SB [9].

<table>
<tr><td></td><td></td><td>one boy’s reflection on whether he will be an independent adult: Maybe not completely, but as good as. . .there is always something that you maybe will see as a problem later in life. I do not really know what now, actually [P7, 17-year- old boy in a wheelchair] SB [9].</td><td></td></tr>
<tr><td></td><td>Biological factors such as bladder and bowel care disruptive to control and routine.</td><td>

<u>Definition:</u> Difficulty at participating in activities, time needed to undertake bladder and bowel care and catheterisation, and physical barriers like incontinence preventing access.

<u>Uniqueness:</u>

<table>
<tr><th></th><th>Proportions [18 Quotes]</th></tr>
<tr><td>Age</td><td>1 x 8-18yrs
2 x 8yrs
1 x 9yrs
2 x 10yrs
1 x 12yrs
1 x 14yrs
1x 15yrs
2 x 16 yrs
4 x 17yrs
2 x 18yrs
1 x unknown</td></tr>
<tr><td>Gender</td><td>Male = 7
Female = 9
Unknown = 2</td></tr>
<tr><td>Condition</td><td>Spinal Bifida = 17
Spinal Cord Injury =1</td></tr>
<tr><td>Parent or Child Interviewed</td><td>Parent = 6
Child = 4
Adolescent = 8</td></tr>
</table>

<u>Quotes:</u>

“[P] really an obstacle...., every 3.5 hour it has to happen.... so you always have to plan ahead, or you have to go back and forth...., you always have to say ‘it’s not possible to come directly after school because he has to go to the toilet first’” [1] Parent, child 8-18yrs, unknown gender, SB</td><td>1, 3, 2, 8, 9</td></tr>
</table>

		"Everything is difficult in my life. I feel tired of living with incontinence and diapers ... I hate the catheterization ... I wondered why children like me with Spina bifida should stay alive." [Girl, 12]. SB [3]<br>Disability-Related Obstacles included: 'bowel program,' [2] caregiver, SCI, unknown gender or age of child<br>'I want her to feel like a big girl and be as much like the rest of them – her friends – as she can' [mother of Caroline, 8 years old] SB.<br>The desire to wear normal underwear was also emphasized by the youth in the study: 'I don't want to be the same like everyone else. The only thing I want to be the same like everyone else is to wear panties' [Talia, 10 years old] SB.<br>On the other hand, some of the older youth such as Ella [15 years old] and Lindsey [18 years old] Female, 18yrs old SB expressed that they were reluctant of using regular underwear, given the uncertain nature of incontinence and the risk of having accidents. Lindsey [18years old] SB on wearing diapers: 'It's like this is my security, I guess...I might try it [wearing regular underwear]; I always keep saying I might try it soon but we'll see' [8].<br><br>I knew that I had to because my parents aren't going to be there in Grade 1 and I just didn't want everybody knowing and I didn't want everybody to be involved in it. I'm just like okay, I'm gonna do it. [Becky, 9 years old] SB [8].<br><br>Parents were very proud when their children were able to independently catheterize and felt this was an important step in the path towards continence:<br>Well, she's been doing [clean intermittent catheterization] since she was six, so she just decided to give it a try and get the mirror out....All it took was putting the idea in Caroline's head and she wanted to try it and well, within a couple of weeks she was doing it all by herself. [mother of Caroline, 8 years old] SB [8].<br><br>Additionally, it was viewed as a barrier to participation when children were unable to manage their incontinence; Landon's father mentioned, 'If he wants to go to a sledge hockey camp overnight and this kind of thing for a week, that's the kind of thing you need to have more independence with'. One mother whose child did not catheterize independently stated that she felt tied to her child because of the need to catheterize her every few hours. Another mother lamented the lack of day camps available to her son owing to his need for support in managing continence [8]. Parent, Male, 16yrs, grade 10, walks with braces, SB L4 myelomeningocele<br><br>Most of the children were able to explain to their peers about spina bifida but left out any information about bowel and bladder control. Danny [17years old] SB stated: '... people know about my disability but I make them think that it's only my legs that are part of it'. [8]. male, SB	

While they were unable to hide the outward physical manifestations of their disability, whenever possible, they kept their continence concerns hidden: 'If I go in there and there are girls in the bathroom I'll walk out just so they don't see my catheter'. [Lindsey, 18 years old] female, SB [8].

However, ultimately, a consistent theme was that embarrassment related to bladder incontinence was seen as the pre-eminent barrier to social participation. For example, when probed about why he felt that his son did not have friends, Landon's father revealed: 'He plays sledge [hockey]. And he does wheelchair racing. He tried basketball. He's been in a kayak. He's done swimming....And yet the friends aren't there. I think it's more of a bowel-bladder issue' [8]. Male, 16yrs, grade 10, walks with braces, SB L4 myelomeningocele

The inability to maintain bladder control also led to some children declining to participate in activities with friends such as swimming or running, because, as Robyn stated, 'whenever I run [my bladder] just lets go'. Many of the children and youth with spina bifida felt that no one at school knew they wore incontinence supplies such as 'pull-ups'. However, parents felt that peers at school were aware of their child's incontinence but tried to maintain their child's dignity by helping them hide the incontinence products [8]. Female, age 10yrs, SB

Negative responses ranged from intrusive questions about toilet regime to down- right bullying behaviour by peers. In response to anticipated negative responses, a strategy of concealing the disability from others was verbalized by one interviewee:
The reason that I have not told my friends is that I see how they are now. Young people, and now I mean people my age, they are kind of, what can I say, immature or they say certain things. They think that if a person is different, then that person is a freak or something like that. That is why I have not dared [to tell them about having SB] [P6, 15-year-old boy walking] SB [9].

The interviewees saw cognitive as well as physical differences between themselves and people without SB.
I probably do not get my homework done as fast as them [peers without SB] and I probably have more difficulties remembering things. And then there is the fact that you have to sneak out sometimes to go to the toilet [P8, 17-year-old boy in a wheelchair] SB [9].

It [using the toilet] does not take more than five or six minutes, so I doubt that I miss very much [of lecture time] [P8, 17-year-old boy using a wheelchair] SB [9].

<table>
<tr><td></td><td></td><td>...but when I was in pre-school the assistant even helped me with peeing and that kind of thing. But then when I started school I had to learn to do it myself, and then the assistant only gave support [with planning schoolwork for example] [P5, 14-year-old girl walking]SB [9].

Participants also described that they did not know how to achieve the desired independence or that they found it difficult to get started. Interviewees harboured intentions to practice the toilet routine with the aim of being able to manage it independently in the future. However, some participants did not have clear ideas about what this practice might actually consist of:
Well, I have to do a bit of checking of the various ways you can do it [manage the toilet] and then practice the one [way of managing the toilet] that seems best, and it should be fine [P7, 17-year-old boy in a wheelchair] SB [9].</td><td></td></tr>
<tr><td></td><td>Needing help from others.</td><td><u>Definition:</u> parents / caregivers / friends / siblings needed to assist with sports, activities of daily living, or bladder care.

<u>Uniqueness:</u>

<table>
<tr><th></th><th>Proportions [Quotes = 9]</th></tr>
<tr><td>Age</td><td>? x unknown
1 x 4-7yrs
1 x 9yrs
2 x 14yrs
2 x 16yrs
1 x 17yrs
1 x 18yrs</td></tr>
<tr><td>Gender</td><td>Male = 4
Female = 3
Unknown = 2</td></tr>
<tr><td>Condition</td><td>Spinal Bifida = 8
Unknown = ?</td></tr>
<tr><td>Parent or Child Interviewed</td><td>Parent = 1
Child = 7
Unknown = ?</td></tr>
</table>

<u>Quotes:</u></td><td>1, 3, 5, 8, 9</td></tr>
</table>

		"[p] the complication is, that the stimulation always has to come from us....what I experience from my healthy children,... is they ask us for help,.... and say 'now you have to help me because ..... I want to do this and that', they ask. He doesn't ask, it always has to be stimulated by us". [1] parent, child 4-7yrs, unknown gender, SB<br><br>Managing university studies alongside a serious health condition and restrictions in mobility can be a daily struggle. I will not be able to study at university, or to work and get married. I'm afraid of what will happen to me if my mother is no longer able to care for me ... who will help me in this miserable life? [Boy, 14] [3]. SB<br><br>'I would like to join a public sports club. I love swimming and playing football with other children ... I am very sad ... It is difficult to reach these areas ... I am not allowed to participate ... I prefer to stay home.' [Boy, 16]. [3]. SB<br><br>Children and adolescents also reported impacts associated with achondroplasia on their daily functioning [Table 1]. The most often mentioned impacts on functioning included the use of adaptive devices [outside school; 100%, n = 32], difficulty reaching objects or high places [84%, n = 27], needing help from others for tasks [outside school; 81%, n = 26],[5] unknown<br><br>'But we always have pushed him to be like other kids, to try to be as much like other kids'. [father of Landon, 16 years old] [8] Male, 16yrs, grade 10, walks with braces, SB L4 myelomeningocele<br><br>I knew that I had to because my parents aren't going to be there in Grade 1 and I just didn't want everybody knowing and I didn't want everybody to be involved in it. I'm just like okay, I'm gonna do it. [Becky, 9 years old] [8]. SB sacral lipomyelomeningocele, Walks independently<br><br>Lindsey [18 years old] Female, SB stated: I was scared of missing something because I was the type of person that was like I want to be here and there and there, and I'm not thinking about going to the bathroom; that didn't like occur to me sometimes. I don't know why. I guess I was a stupid little kid. [8] SB L4 lipomyelomeningocele, walks with braces and a crutch<br><br>...but when I was in pre-school the assistant even helped me with peeing and that kind of thing. But then when I started school I had to learn to do it myself, and then the assistant only gave support [with planning schoolwork for example] [P5, 14-year-old girl walking] [9].<br><br>one boy's reflection on whether he will be an independent adult: Maybe not completely, but as good as. . .there is always something that you maybe will see as a problem later in life. I do not really know what now, actually [P7, 17-year- old boy in a wheelchair] [9].	

<table>
<tr>
<td>Psychological Factors</td>
<td>Feeling don’t fit in. Adolescents feeling rejected by peers.</td>
<td>
<u>Definition:</u> awareness of difference from peers and not fitting in. Being rejected or bullied by peers, particularly around continence issues. Parents / caregivers hiding their child. Desire to be like everyone else and transfer to disability school. Lack of real friendships despite being ‘friends’ on social media.

<u>Uniqueness:</u>

<table>
<tr><th></th><th>Proportions [Quotes = 20]</th></tr>
<tr><td>Age</td><td>2 x Unknown
1 x 8yrs
7 x 10yrs
2 x 12yrs
2 x 14yrs
2 x 16yrs
2 x 17yrs
2 x 18yrs</td></tr>
<tr><td>Gender</td><td>Male = 5
Female = 14
Unknown = 1</td></tr>
<tr><td>Condition</td><td>Spinal Bifida = 19
Spinal Cord Injury = 1</td></tr>
<tr><td>Parent or Child Interviewed</td><td>Parent = 4
Child = 16</td></tr>
</table>

<u>Quotes:</u>

My child is somewhat shy and not particularly outgoing. This was also true prior to his injury,’ and ‘feeling that he ‘cannot do that’, or ‘would not fit in’. [2]. Parent, unknown gender, unknown age, SCI

Some children expressed their desire to be transferred to a special disability school to feel normal or have friends.
The school took me out of my comfort zone ... I became aware that I'm different from some children who stared at me and made fun of my shaky walk. [Girl, 12]. [3]. SB
</td>
<td>2, 3, 4, 8, 9</td>
</tr>
</table>

		Participants offered functional suggestions of how to improve the lives of people living with SB, such as expanding wheelchair accessibility in public spaces and raising awareness and acceptance of SB:<br>“Yes, I do think that if it’s put out there and the media is aware of it that it would change things. Because I find that people tend to hide their kids” [P5, 42-year-old female]. [4]. SB<br><br>‘Sometimes it gets into my head and then I start thinking that I’m different and that – like I start believing them’ [Robyn, 10 years old]. [8] female, SB<br><br>Landon stated: ‘I just don’t want to be judged because I’m different’. [8]. Male, 16yrs, grade 10, walks with braces, SB L4 myelomeningocele<br><br>Jasmin, 14 years old, acknowledged the difficult process she had experienced in accepting her identity as someone with a disability: ‘I went through very many emotional roller coasters to finally realise that I was not like everyone else and I had to accept that’. [8] Jasmin, female, 14yrs, uses wheelchair, SB L4 lipomyelomeningocele<br><br>‘I want her to feel like a big girl and be as much like the rest of them – her friends – as she can’ [mother of Caroline, 8 years old]. SB<br><br>The desire to wear normal underwear was also emphasized by the youth in the study: ‘I don’t want to be the same like everyone else. The only thing I want to be the same like everyone else is to wear panties’ [Talia, 10 years old] [8]. SB<br><br>Parents feared that other children would smell the urine and that this would lead to ridicule from their peers [reports from some children suggested that this did happen] [8].<br><br>Some of the older youth expressed that they wish they had been able to achieve greater independence with CIC when they were younger as it would have facilitated greater social participation and friendships.<br>Lindsey [18 years old] Female, SB stated: I was scared of missing something because I was the type of person that was like I want to be here and there and there, and I’m not thinking about going to the bathroom; that didn’t like occur to me sometimes. I don’t know why. I guess I was a stupid little kid [8]. SB<br><br>SB Danny [17years old] stated: ‘... people know about my disability but I make them think that it’s only my legs that are part of it’. male, SB	

		While they were unable to hide the outward physical manifestations of their disability, whenever possible, they kept their continence concerns hidden: 'If I go in there and there are girls in the bathroom I'll walk out just so they don't see my catheter'. [Lindsey, 18 years old] [8]. Female SB<br><br>Robyn [10 years old] identified that this was often specifically associated with her incontinence: 'They make fun of me and they say like, "Oh, you stink"' [8]. Female, SB<br><br>Michael reported that he played mainly with his younger brother's friends, [8]. Male, 10yrs old, SB<br><br>Talia's mother discussed that her daughter's friends were her cousins and kids from the neighbourhood who wanted to hang out at her house even when Talia was not home. [8]. Parent, female, 10yrs old, SB<br><br>Danny [17 years old] stated that he did not have many friends but seemed to accept the situation: 'It doesn't matter if I'm hanging out with friends or if I'm by myself, I don't care either way. I'm sort of always happy so it doesn't really matter to me' [8]. male, SB<br><br>Jasmin [14years old] had some close friends but felt that her peers had difficulty seeing beyond her mobility impairments: 'People they just kind of like, they look at me and they are like...okay...the chair is all they see'. [8]. Jasmin, female, 14yrs, uses wheelchair, SB L4 lipomyelomeningocele<br><br>Robyn mentioned: 'Some of the friends I have [on Facebook] they'll send me a friend request and I'll say yes, but...when I go to school it's like they don't even know I exist or they don't even talk to me'.[8]. Female, 10yrs old, SB<br><br>One participant stated that he did not feel comfortable going to other people's houses because of his need to catheterize. He stated, 'Because if they see what's in the garbage, then they'll ask me questions. I don't really want to explain it to them' [Landon, 16years old] [8]. Male, 16yrs, grade 10, walks with braces, SB L4 myelomeningocele<br><br>those [persons with SB] that I have met have had a lot more problems than me, with difficulties in walking at all [not audible], so I think I am one of those that are less affected [P3, 12-year-old girl walking]. [9]. SB	

<table>
<tr><td></td><td></td><td>For example, one participant identified herself as being similar to peers without disabilities based on experiences when playing in a park area:
Well, I can join them. I can move pretty fast, although not as fast [as the peers] [P1, 10-year-old girl using ambulatory devices/wheelchair]. [9]. SB</td><td></td></tr>
<tr><td></td><td>Anger towards inability to participate in school activities and social events.</td><td>

Definition: Positive and negative emotions towards ability / inability to participate in school activities.

Uniqueness:

<table>
<tr><th></th><th>Proportions [Quotes = 10]</th></tr>
<tr><td>Age</td><td>Unknown = 2
2 x 10yrs
1 x 14yrs
2 x 16yrs
1 x 16yrs
1 x 17yrs
1 x 18yrs</td></tr>
<tr><td>Gender</td><td>Male = 4
Female = 4
Unknown = 2</td></tr>
<tr><td>Condition</td><td>Spinal Bifida = 8
Spinal Cord Injury = 1
Post-op Scoliosis Surgery = 1</td></tr>
<tr><td>Parent or Child Interviewed</td><td>Parent = 1
Caregiver = 1
Child = 8</td></tr>
</table>

Quotes:

‘PE [physical education] teacher in high school did not think she could participate in PE although she had been doing PE since fifth grade, so they put her in marching band.’ [2]. Caregiver, Unknown age, unknown gender, SCI

I was excluded from all the outings, such as field trips and physical education classes ... I like to play football. But I couldn't participate in that ... The teachers said ‘It is difficult. You just sit there and watch the other children. [Boy, 14]. [3]. SB
</td><td>2, 3, 6, 7, 8, 9</td></tr>
</table>

		I would like to join a public sports club. I love swimming and playing football with other children ... I am very sad ... It is difficult to reach these areas ... I am not allowed to participate ... I prefer to stay home. [Boy, 16]. [3]. SB<br><br>Well I had a really great teacher so she would always include me in everything. She modified things when I got tired. I remember one time I got to play ping pong with people instead. [Nelly] [6]. Female, 16yrs, SB SB<br><br>I go to the gym at school ... I usually work on the punching bag, weight stuff like that. I did take fitness course and I took gym for a while. As well as I saw my friends doing it and I guess I started to imitate them. [Huessein] [6]. Male, 17yrs SB<br><br>When asked about how they felt not being able to participate in sports and activity for a period of time after surgery, participants reported feeling frustrated, and reported that they missed playing on their sports teams: "It sucked for a while; I was a pretty active person so the first 6–7 months kind of sucked". [7]. Child, unknown age, unknown gender, post-op scoliosis surgery<br><br>Additionally, it was viewed as a barrier to participation when children were unable to manage their incontinence; Landon's father mentioned, 'If he wants to go to a sledge hockey camp overnight and this kind of thing for a week, that's the kind of thing you need to have more independence with'. [8] Male, 16yrs, grade 10, walks with braces, SB L4 myelomeningocele<br><br>Lindsey [18 years old] stated: I was scared of missing something because I was the type of person that was like I want to be here and there and there, and I'm not thinking about going to the bathroom; that didn't like occur to me sometimes. I don't know why. I guess I was a stupid little kid. [8]. Female SB<br><br>Robyn stated, 'whenever I run [my bladder] just lets go'. [8]. Female, 10yrs old, SB<br><br>Well, I can join them. I can move pretty fast, although not as fast [as the peers] [P1, 10-year-old girl using ambulatory devices/wheelchair] [9]. SB	
	Religious beliefs being source of hope.	**Definition:** faith, belief and religion being a source of hope and reconciliation.<br><br>**Uniqueness:**	3, 4

<table>
<tr>
<td></td>
<td></td>
<td>
<table>
<tr><th></th><th>Proportions [Quotes = 4]</th></tr>
<tr><td>Age</td><td>Unknown = 1
1 x 7yrs
1 x 13yrs
1 x 16yrs</td></tr>
<tr><td>Gender</td><td>Male = 1
Female = 3</td></tr>
<tr><td>Condition</td><td>Spinal Bifida = 3</td></tr>
<tr><td>Parent or Child Interviewed</td><td>Parent = 0
Child = 4</td></tr>
</table>
<u>Quotes:</u>
My mother taught me that I am a gift from God. She told me I will walk in heaven and will gain later whatever I have lost in this life. [Girl, 7]. [3] SB
The religious beliefs I learned from my family and from my mum still help me. My parents took me to Saudi Arabia to request prayers ... to protect me and my family. [Boy, 16] [3] SB
"God had chosen them as a special child" [P3, age unknown female]. [4] SB
"I'm grateful that I've got family, great friends...I'm grateful because God created me, I'm unique. Thankful for the love of my family, what they gave ... " [P1, 13-year-old female]. [4] SB
</td>
<td></td>
</tr>
<tr>
<td>Social factors which impact access to PA</td>
<td>[a] emotions like fear and worry</td>
<td>
<u>Definition:</u> fear and worry being obstacles to socialisation and participation. Fearful about injury, fearful about harm from others in public places, fearful about negativity from others or ridicule. Fear about inability to do things.
<u>Uniqueness:</u>
<table>
<tr><th></th><th>Proportions [Quotes = 9]</th></tr>
<tr><td>Age</td><td>Unknown = 4
2 x 13
1 x 14
1 x 15
1 x 16</td></tr>
</table>
</td>
<td>2, 4, 7, 8, 9</td>
</tr>
</table>

**Gender**	Male = 2<br>Female = 4<br>Unknown = 3
**Condition**	Spinal Bifida = 6<br>Spinal Cord Injury = 2<br>Post-op Scoliosis = 1
**Parent or Child Interviewed**	Caregiver = 2<br>Child = 7

Quotes:

Other Obstacles included: 'weather [winter bad roads],' 'too young,' and 'the fear of getting more injuries.' [2]. Caregiver, Unknown age, unknown gender, SCI

'he has friends but I do not feel that the kids would encourage my child to play on their sports team; they would feel that it may make them lose,' 'people worry that he will get hurt and they will be liable,' and 'PE [physical education] teacher in high school did not think she could participate in PE although she had been doing PE since fifth grade, so they put her in marching band.' [2] Caregiver, Unknown age, unknown gender, SCI

"I'm just worried about her health because ... she's don't know, looks like [adolescent participant] don't see the importance of her stuff that she must do" [P3, age unknown female]. [4] SB

"Of those killing, killing kids, girls, taking girls and grabbing them and killing taking the body parts. I don't go outside anymore. I never go outside" [P1, 13-year-old female]. [4] SB

"We don't go out like to the mall, we just see each other at school and on the streets" [P1, 13-year-old female]. [4] SB

"I'm really scared about getting hurt." [7] Child, Unknown age, unknown gender, Post-op scoliosis surgery

Landon's father revealed: 'He plays sledge [hockey]. And he does wheelchair racing. He tried basketball. He's been in a kayak. He's done swimming....And yet the friends aren't there. I think it's more of a bowel-bladder issue' [8]. Male, 16yrs, grade 10, walks with braces, SB L4 myelomeningocele

<table>
<tr>
<td></td>
<td></td>
<td>The reason that I have not told my friends is that I see how they are now. Young people, and now I mean people my age, they are kind of, what can I say, immature or they say certain things. They think that if a person is different, then that person is a freak or something like that. That is why I have not dared [to tell them about having SB] [P6, 15-year-old boy walking] [9]. SB

The thing is that I am not good at mathematics and I have noticed that you should be [to manage the desired occupation] [laughter]. I have some difficulties with mathematics, and then [. . .] I have difficulties remembering things, like I said, but I will probably have to. . . I have to practice so that I can remember [...] Writing things down, I have to pull myself together on that [laughter]. It is this kind of thing that I might worry a bit about that it will not work out [P5, 14-year-old girl walking] [9]. SB</td>
<td></td>
</tr>
<tr>
<td></td>
<td>[c] dependency of child and impact on routine [work, family, time],</td>
<td><u>Definition:</u> desire to be independent and requirement for caregiver / parent / sibling support with activities, toileting, pushing wheelchair.

<u>Uniqueness:</u>

<table>
<tr><th></th><th>Proportions [Quotes = 12]</th></tr>
<tr><td>Age</td><td>1 x unknown
2 x 4-7yrs
1 x 7yrs
3 x 8-18yrs
1 x 13yrs
1 x 14yrs
1 x 16yrs
2 x 17yrs</td></tr>
<tr><td>Gender</td><td>Male = 5
Female = 1
Unknown = 6</td></tr>
<tr><td>Condition</td><td>Spinal Bifida = 12</td></tr>
<tr><td>Parent or Child Interviewed</td><td>Parent = 2
Caregiver = 1
Child = 9</td></tr>
</table>

<u>Quotes:</u></td>
<td>1, 3, 4, 9</td>
</tr>
</table>

		"[C] I always self-propel my wheelchair,....at a certain point,... you have to do it yourself later on". [1] Child age 8-18yrs , unknown gender, SB<br><br>"[p] the complication is, that the stimulation always has to come from us....what I experience from my healthy children,... is they ask us for help,.... and say 'now you have to help me because ..... I want to do this and that', they ask. He doesn't ask, it always has to be stimulated by us". [1]. Parent, age 4-7yrs , unknown gender, SB<br><br>"[C] I think partly maybe the way I was brought up, because my parents they always say, you have to propel yourself as much as possible, because your fitness will increase......if I start to complain 'I'm tired, I want to go home', well, they ignore it......I think it is ok, I think I will benefit later on. On the other hand, at that moment when I'm tired and they don't want to push me, I am mad [laughing]". [1]. Child age 8-18yrs , unknown gender, SB<br><br>"[C] It has to be in the neighborhood....so you can go by yourself...so your parents don't have to take you. Yes, because when you grow older, it is annoying always having your parents around". [1] Child age 8-18yrs , unknown gender, SB<br><br>"[p] there are a lot of enthusiastic people who said it really fits in our club, and we're going to take care of it!". [1]. Parent, age 4-7yrs , unknown gender, SB<br><br>My family does everything for me. I don't have to do anything. I just play and study. [Girl, 7]. [3] SB<br><br>Managing university studies alongside a serious health condition and restrictions in mobility can be a daily struggle. I will not be able to study at university, or to work and get married. I'm afraid of what will happen to me if my mother is no longer able to care for me ... who will help me in this miserable life? [Boy, 14] [3]. SB<br><br>I would like to join a public sports club. I love swimming and playing football with other children ... I am very sad ... It is difficult to reach these areas ... I am not allowed to participate ... I prefer to stay home. [Boy, 16]. [3]. SB<br><br>"We have done so many things, trying to inculcate in her she needs to do what she's supposed to be doing all the time. She needs to be consistent" [P2, 43-year-old male]. [4]. Unknown gender, unknown age, SB	

<table>
<tr><td></td><td></td><td>“Cause I don’t wanna be like in a wheelchair. It’s not fun to be in. Then I can do nothing, like say my now I wanna go play soccer then I can’t cause I’m here in. And then I can’t do what other children can do” [P8, 13-year-old male]. [4]. SB

There are not a lot of things that can worry me. It is like, I improvise. If anything comes up that I cannot solve, we [participant and parents] will improvise. Then we will solve it when the problem arises [P8, 17-year-old boy in a wheelchair] [9]. SB

Well, I have to do a bit of checking of the various ways you can do it [manage the toilet] and then practice the one [way of managing the toilet] that seems best, and it should be fine [P7, 17-year-old boy in a wheelchair] [9]. SB</td><td></td></tr>
<tr><td></td><td>[d] for some a need to adjust and accept</td><td><u>Definition:</u> Need to adjust and accept. Feeling upset / miserable / depressed / negative towards physical condition. Lack of awareness in general population regarding condition. Joining a sports team a positive step towards acceptance and living with condition.

<u>Uniqueness:</u>

<table>
<tr><th></th><th>Proportions [Quotes = 11]</th></tr>
<tr><td>Age</td><td>Unknown = 5
1 x 12yrs
1 x 15yrs
1 x 10yrs
2 x 14yrs
1 x 17yrs</td></tr>
<tr><td>Gender</td><td>Male = 3
Female = 3
Unknown = 5</td></tr>
<tr><td>Condition</td><td>Spinal Bifida = 11</td></tr>
<tr><td>Parent or Child Interviewed</td><td>Caregiver = 5
Parent = 1
Child = 5</td></tr>
</table>

<u>Quotes:</u>

“Yes just they mustn’t be shy for their child ... The people must adjust around them, and the families must try to adapt to how they are man. They mustn’t feel out because of their disability” [P11, 45-year- old female]. [4]. SB, unknown age, unknown gender</td><td>3, 4, 8, 9</td></tr>
</table>

		Three years ago, I felt depressed. I tried to end my life ... Then my parents talked to me and helped me continue this miserable life. [Girl, 12]. [3]. SB<br><br>"Yes just they mustn't be shy for their child ... The people must adjust around them, and the families must try to adapt to how they are man. They mustn't feel out because of their disability" [P11, 45-year- old female]. [4]. SB, unknown age, unknown gender<br><br>"Tell them to join sports, that's all I can tell. I think, first I didn't want to do athletics, now I can beat everyone." [P13, 15-year-old male]. [4] SB<br><br>"Yes, I do think that if it's put out there and the media is aware of it that it would change things. Because I find that people tend to hide their kids" [P5, 42-year-old female]. [4] SB, unknown age, unknown gender<br><br>"You know, so a lot of people know nothing about spina bifida. A lot of people even our families they have no idea. And even when they see children who look like ... they have no idea and yet there are so many people even in our country living with spina bifida" [P2, 43-year- old male]. [4] SB, unknown age, unknown gender<br><br>"He's not happy at the moment. I don't know when he will accept it. But he is not happy at all" [P14, 75-year-old male]. [4] SB, unknown age, unknown gender<br><br>Michael's father stated: '[He's] just a regular, everyday kid'…. However, as the interviews progressed, it was clear that most parents did not really see their children as a 'typical' kid, as with probing, they cited considerable differences between their child with spina bifida and other children [e.g. their siblings] in domains such as peer relationships, academic success and social participation [8]. Male, 10yrs old, SB<br><br>Jasmin, 14 years old, 'I went through very many emotional roller coasters to finally realise that I was not like everyone else and I had to accept that' [8]. Jasmin, female, 14yrs, uses wheelchair, SB L4 lipomyelomeningocele	

<table>
<tr>
<td></td>
<td></td>
<td>
There are not a lot of things that can worry me. It is like, I improvise. If anything comes up that I cannot solve, we [participant and parents] will improvise. Then we will solve it when the problem arises [P8, 17-year-old boy in a wheelchair] [9].

The thing is that I am not good at mathematics and I have noticed that you should be [to manage the desired occupation] [laughter]. I have some difficulties with mathematics, and then [. . .] I have difficulties remembering things, like I said, but I will probably have to. . . I have to practice so that I can remember [...] Writing things down, I have to pull myself together on that [laughter]. It is this kind of thing that I might worry a bit about that it will not work out [P5, 14-year-old girl walking] [9].
</td>
<td></td>
</tr>
<tr>
<td></td>
<td>Children / adolescents feeling ashamed and experiencing peers hurtful behaviour / bullying. Negative attitudes and social acceptance.</td>
<td>
<u>Definition:</u> negative attitudes and behaviour from peers, lacking social acceptance. Keeping aspects of their health hidden / private to avoid ridicule.

<u>Uniqueness:</u>

<table>
<tr><th></th><th>Proportions [Quotes = 16]</th></tr>
<tr><td>Age</td><td>2 x Unknown + ??
1 x 9yrs
3 x 10yrs
1 x 12yrs
2 x 14yrs
2 x 15yrs
3 x 16yrs
1 x 17yrs
1x 18yrs</td></tr>
<tr><td>Gender</td><td>Male = 10
Female = 4
Unknown = 1 + ?</td></tr>
<tr><td>Condition</td><td>Spinal Bifida = 15
Achondroplasia = ?</td></tr>
<tr><td>Parent or Child Interviewed</td><td>Caregiver = 1
Child = 14
Unknown = ?</td></tr>
</table>

<u>Quotes:</u>
</td>
<td>1, 3, 4, 6, 8, 9</td>
</tr>
</table>

		Managing university studies alongside a serious health condition and restrictions in mobility can be a daily struggle. I will not be able to study at university, or to work and get married. I'm afraid of what will happen to me if my mother is no longer able to care for me ... who will help me in this miserable life? [Boy, 14] [3] SB<br><br>I hate school ... I feel that many children give me looks and put me down ... because they discovered my incontinence ... They always make me feel afraid and ashamed ... I usually like to stay alone. [Boy, 10]. [3] SB<br><br>The school took me out of my comfort zone ... I became aware that I'm different from some children who stared at me and made fun of my shaky walk. [Girl, 12]. [3] SB<br><br>I prefer to stay home ... You know how people in our society deal with such cases ... they look at me curiously ... I do not like to hear words of sympathy ... It's really hard ... I'm a human. And I want people to respect me. [Boy, 16]. [3] SB<br><br>My friends, mainly cousins, are very helpful. I am happy to have them in my life ... They often encourage me. I'm not allowed to stay out with them for a long period ... but I'm lucky that I can keep contact with them through the computer. [Boy, 15]. [3] SB<br><br>I pretend to use the bathroom while I am at school ... So that other children think I'm normal and can use the bathroom as they do. [Boy, 10]. [3] SB<br><br>My brothers are similar to me. The only difference is that they can walk. But I can crawl and control the wheelchair. [Boy, 9]. [3] SB<br><br>"I think the only good influences that she has are the ones that are in the neighbourhood, because ... I don't know they not negative ... " [P2, 43-year-old male]. [4]. Caregiver, unknown age, unknown gender, SB<br><br>being stigmatized by others [38%, n = 12], peers treating them differently [e.g., being protective of child, 31%, n = 10], and positive impacts on friendships [e.g., making friends easily, 28%, n = 9]. [5] Achondroplasia, unknown ages , unknown genders<br><br>'Sometimes it gets into my head and then I start thinking that I'm different and that – like I start believing them' [Robyn, 10 years old]. [8] female, SB	

<table>
<tr>
<td></td>
<td></td>
<td>Landon stated: ‘I just don’t want to be judged because I’m different’. [8] Male, 16yrs, grade 10, walks with braces, SB L4 myelomeningocele

Jasmin, 14 years old, ‘I went through very many emotional roller coasters to finally realise that I was not like everyone else and I had to accept that’ [8]. Jasmin, female, 14yrs, uses wheelchair, SB L4 lipomyelomeningocele

Danny [17years old] stated: ‘... people know about my disability but I make them think that it’s only my legs that are part of it’. [8] male, SB

‘If I go in there and there are girls in the bathroom I’ll walk out just so they don’t see my catheter’. [Lindsey, 18 years old] [8]. Female, SB

‘Because if they see what’s in the garbage, then they’ll ask me questions. I don’t really want to explain it to them’ [Landon, 16years old] [8]. Male, 16yrs, grade 10, walks with braces, SB L4 myelomeningocele

The reason that I have not told my friends is that I see how they are now. Young people, and now I mean people my age, they are kind of, what can I say, immature or they say certain things. They think that if a person is different, then that person is a freak or something like that. That is why I have not dared [to tell them about having SB] [P6, 15-year-old boy walking] [9].</td>
<td></td>
</tr>
<tr>
<td></td>
<td>Friends being a source of needed emotional support and role models.</td>
<td><u>Definition:</u> having a friendship group as a source of social inclusion.

<u>Uniqueness:</u>
<table>
<tr><th></th><th>Proportions [Quotes = 12]</th></tr>
<tr><td>Age</td><td>1 x unknown + ?
1 x 8-18yrs
2 x 8yrs
4 x 9yrs
1 x 15yrs
2 x 18yrs</td></tr>
<tr><td>Gender</td><td>Male = 1
Female = 9
Unknown = 1 + ?</td></tr>
</table>
</td>
<td>1, 3, 4, 5, 6, 8</td>
</tr>
</table>

**Condition**	Spinal Bifida = 11<br>Achondroplasia = ?
**Parent or Child Interviewed**	Parent = 2<br>Caregiver = 1<br>Child = 8<br>Unknown = ?

Quotes:

"[C] because you're around people, you make contact with people, sometimes you make friends". In the children and adolescents however, was also mentioned that PA was not important. [1] Child age 8-18yrs , unknown gender, SB

My friends, mainly cousins, are very helpful. I am happy to have them in my life ... They often encourage me. I'm not allowed to stay out with them for a long period ... but I'm lucky that I can keep contact with them through the computer. [Boy, 15]. [3] SB

"I don't know, [adolescent participant]...if she gets um supported by her friends at school because she was she was usually the child that was bullied at school. Because I think it was two years ago, not last year, but she came home with her eye with a blue eye that one of the children did hit her. So I'm not sure" [P3, age unknown female]. [4] caregiver, SB

positive impacts on friendships [e.g., making friends easily, 28%, n = 9]. [5] Achondroplasia, unknown genders, unknown ages

Friends and teammates also had a positive influence on the youths' PA behaviour by providing emotional support [e.g., applauding their effort at sports games] and companionship [i.e., doing a strength-training workout over video chat together]. [6] SB

Caroline [8 years old], Becky [9 years old] and Lindsey [18 years old] Female, SB reported that they had many close friends, with

Becky discussing weekly sleepovers with friends and female, 9yrs old, SB

Lindsey revealing a consistent group of close friends that she spent time with every weekend. Female, 18yrs old SB

<table>
<tr>
<td></td>
<td></td>
<td>Parents of Caroline and Becky also reported that they were accepted by their peers, regardless of their disability. Becky female, 9yrs old, SB, Caroline 8yrs old, female, SB

Becky was very upfront with her friends about her need to catheterize: ‘So I just decided to tell them before they found out and got shocked and ran away screaming’ [8]. female, 9yrs old, SB</td>
<td></td>
</tr>
<tr>
<td></td>
<td>Youth dependent on parents for advice or support and fear avoidance.</td>
<td><u>Definition:</u> dependence on parents for physical and emotional support. Parents supporting child with catheterisation, sports and physical functioning.

<u>Uniqueness:</u>

<table>
<tr><th></th><th>Proportions [Quotes = 13]</th></tr>
<tr><td>Age</td><td>2 x unknown
2 x 7yrs
1 x 8-18yrs
1 x 8yrs
1 x 9yrs
1 x 10yrs
1 x 12yrs
2 x 14yrs
1 x 16yrs
1 x 17yrs</td></tr>
<tr><td>Gender</td><td>Male = 3
Female = 7
Unknown = 3</td></tr>
<tr><td>Condition</td><td>Spinal Bifida = 12
Post-op Scoliosis = 1</td></tr>
<tr><td>Parent or Child Interviewed</td><td>Parent = 4
Caregiver = 0
Child = 9</td></tr>
</table>

<u>Quotes:</u>

“[P] really an obstacle...., every 3.5 hour it has to happen.... so you always have to plan ahead, or you have to go back and forth...., you always have to say ‘it’s not possible to come directly</td>
<td>1, 3, 4, 6, 7, 8, 9</td>
</tr>
</table>

		after school because he has to go to the toilet first', it is even a bigger obstacle than the handicap, you always have to be there as a parent..." [1] Parent age 8-18yrs , unknown gender, SB<br><br>Three years ago, I felt depressed. I tried to end my life ... Then my parents talked to me and helped me continue this miserable life. [Girl, 12]. [3] SB<br><br>My family does everything for me. I don't have to do anything. I just play and study. [Girl, 7]. [3] SB<br><br>Managing university studies alongside a serious health condition and restrictions in mobility can be a daily struggle. I will not be able to study at university, or to work and get married. I'm afraid of what will happen to me if my mother is no longer able to care for me ... who will help me in this miserable life? [Boy, 14] [3] SB<br><br>Paralysis makes my situation tragic ... I was just sitting near the heater. I did not notice that my legs were burnt until my mother entered the room and started shouting. [Boy, 14]. [3] SB<br><br>My mother taught me that I am a gift from God. She told me I will walk in heaven and will gain later whatever I have lost in this life. [Girl, 7]. [4] SB<br><br>Well my mom just signed me up for para swimming ... I didn't want to, my mom forced me. [Nelly] [6] Female, 16yrs, SB<br><br>The process of deciding whether to do an activity included steps: *"I talk to my mom and my dad but then also, if I'm doing something and its hurting or whatever I just stop. Or if I'm not really too sure about something, I'll do a little bit... or lift a portion of it if that's possible, and then if I'm okay I just kind of keep going and then if it hurts, I stop immediately."* Pain led to stopping an activity, and fear was a factor that created avoidance behaviour and kept participants from trying allowed activities: *"I'm really scared about getting hurt." [7]* Child, unknown age, unknown gender, post-op scoliosis surgery<br><br>I knew that I had to because my parents aren't going to be there in Grade 1 and I just didn't want everybody knowing and I didn't want everybody to be involved in it. I'm just like okay, I'm gonna do it. [Becky, 9 years old] [8]. SB<br><br>Well, she's been doing [clean intermittent catheterization] since she was six, so she just decided to give it a try and get the mirror out....All it took was putting the idea in Caroline's head and	

<table>
<tr>
<td></td>
<td></td>
<td>she wanted to try it and well, within a couple of weeks she was doing it all by herself. [mother of Caroline, 8 years old] [8] SB

Robyn’s father expressed this frustration:
It was a goal we were working towards....make her independent to make her take ownership of her situation, and if you do, this is what you could have, you get...a very small absorbent pad versus a pull up. So it worked for a little while and then she fell off the wagon. [8] Female, age 10yrs, SB

One mother whose child did not catheterize independently stated that she felt tied to her child because of the need to catheterize her every few hours. Another mother lamented the lack of day camps available to her son owing to his need for support in managing continence [8].SB, unknown age, unknown gender

There are not a lot of things that can worry me. It is like, I improvise. If anything comes up that I cannot solve, we [participant and parents] will improvise. Then we will solve it when the problem arises [P8, 17-year-old boy in a wheelchair] [9]. SB</td>
<td></td>
</tr>
<tr>
<td></td>
<td>Lacking specific information re PA and FITT.

Lack of support from sports counsellors, local clubs or organisations.</td>
<td><u>Definition:</u> Information available for children / adolescents with spinal conditions. Lack of information from HCPs, teachers, school or sports coaches. Leading to lack of awareness in child / adolescent and caregiver regarding activities.

<u>Uniqueness:</u>

<table>
<tr><th></th><th>Proportions [Quotes = 10]</th></tr>
<tr><td>Age</td><td>4 x unknown
1 x 4-7yrs
3 x 8-18yrs
1 x 15yrs
1 x 17yrs</td></tr>
<tr><td>Gender</td><td>Male = 1
Female = 1
Unknown = 8</td></tr>
<tr><td>Condition</td><td>Spinal Bifida = 9
Spinal Cord Injury =1</td></tr>
<tr><td>Parent or Child Interviewed</td><td>Parent = 4
Caregiver = 4</td></tr>
</table>
</td>
<td>1, 2, 4, 6</td>
</tr>
</table>

		<table><tr><td></td><td>Child = 2</td></tr></table><br><br>**<u>Quotes:</u>**<br><br>"[P] A lot of things you have to find out yourself...I do miss that.....I think, if you're in a hospital, we visit the hospital regularly, that there should be.....more information...and listening what the child wants and I do miss that......they ask for example 'how is it', 'yes everything goes well' he [the child] says, well he always says everything goes well......but I think....you should ask 'what else do you want, how is it going with playing sports, do you play sports', it is always about what school do you go to and that's that". [1] Parent age 8-18yrs , unknown gender, SB<br><br>"[p] especially one on one, somebody who says.....that's all available, what kind of child do you have, what kind of situation, where do you live, what are you looking for, leading to something concrete". [1] Parent age 4-7yrs , unknown gender, SB<br><br>"[P] In third grade they spent a lesson on him, they have this book, ...it is about a boy with SB....the teacher read it aloud and then they talked about it", [1] Parent age 8-18yrs , unknown gender, SB<br><br>"[P] we always had support from a regional expertise centre and a therapist comes in ones every so many times". [1] Parent age 8-18yrs , unknown gender, SB<br><br>'lack of knowledge in the community,' and 'there are not enough local community activities that can include someone with a physical disability ... most programming is geared towards individuals with cognitive disabilities.' [2]. Caregiver, unknown age, unknown gender, SCI<br><br>"There's no support... So we're trying to do that, like maybe on one occasion we have like maybe the, the session for the boys, where they'll do shaving, or you know, self-care" [P5, 42-year-old female]. [4] unknown age, unknown gender, SB<br><br>"Yes, I do think that if it's put out there and the media is aware of it that it would change things. Because I find that people tend to hide their kids" [P5, 42-year-old female]. [4] unknown age, unknown gender, SB<br><br>"You know, so a lot of people know nothing about spina bifida. A lot of people even our families they have no idea. And even when they see children who look like ... they have no idea and yet there are so many people even in our country living with spina bifida" [P2, 43-year- old male]. [4]. unknown age, unknown gender, SB	

<table>
<tr><td rowspan="2"></td><td></td><td>[my coaches] have been super supportive and understanding. They adjust accordingly, and if I have to leave the ice they let the ref know and the game know. They accommodate and adjust. [Jennifer] [6] Female, 15yrs old, SB

they had not been given any specific instructions regarding the type of activity to undertake or how often to be physically active in order to meet the PA guidelines [Knowledge].
[PA duration] mmm ... around an hour I think ... maybe a little more. [Hussein] [6]. Male, 17yrs, Manual Wheelchair, SB</td><td></td></tr>
<tr><td>Desire to be active and increase independence self-propelling wheelchair.</td><td><u>Definition:</u> desire to be active and independent. Recognition of physical ability as a facilitator of social independence [self-propelling wheelchair].

<u>Uniqueness:</u>

<table>
<tr><th></th><th>Proportions [Quotes = 12]</th></tr>
<tr><td>Age</td><td>1 x unknown
2 x 8-18yrs
2 x 10yrs
2 x 12yrs
1 x 13yrs
2 x 14yrs
1 x 16yrs
1 x 17yrs</td></tr>
<tr><td>Gender</td><td>Male = 5
Female = 4
Unknown = 3</td></tr>
<tr><td>Condition</td><td>Spinal Bifida = 12</td></tr>
<tr><td>Parent or Child Interviewed</td><td>Parent = 2
Caregiver = 3
Child = 8</td></tr>
</table>

<u>Quotes:</u></td><td>1, 4, 8, 9</td></tr>
</table>

		Wanting to be physically active and to be independent seems to be a very strong positive theme; "[C] I always self-propel my wheelchair,....at a certain point,... you have to do it yourself later on". [1] Child age 8-18yrs , unknown gender, SB<br><br>"[C] It has to be in the neighborhood....so you can go by yourself...so your parents don't have to take you. Yes, because when you grow older, it is annoying always having your parents around". [1] Parent age 8-18yrs , unknown gender, SB<br><br>I will not be able to study at university, or to work and get married. I'm afraid of what will happen to me if my mother is no longer able to care for me ... who will help me in this miserable life? [Boy, 14]. [3] SB<br><br>"We have done so many things, trying to inculcate in her she needs to do what she's supposed to be doing all the time. She needs to be consistent" [P2, 43-year-old male]. [4] SB<br><br>"Cause I don't wanna be like in a wheelchair. It's not fun to be in. Then I can do nothing, like say my now I wanna go play soccer then I can't cause I'm here in. And then I can't do what other children can do" [P8, 13-year-old male]. [4] SB<br><br>'She participates at school like any other child and participates in extracurricular activities like any other child and is treated like anyone else' [father of Robyn, 10 years old]. [8] Female, SB<br><br>One father mentioned that he pushed his son to walk instead of using a wheelchair, even though this often resulted in blisters because of the lack of sensation in his feet: 'But we always have pushed him to be like other kids, to try to be as much like other kids'. [father of Landon, 16 years old] [8]. Male, 16yrs, grade 10, walks with braces, SB L4 myelomeningocele<br><br>Jasmin [14years old] had some close friends but felt that her peers had difficulty seeing beyond her mobility impairments: 'People they just kind of like, they look at me and they are like...okay...the chair is all they see' [8]. Jasmin, female, 14yrs, uses wheelchair, SB L4 lipomyelomeningocele<br><br>[laughter] I am quite good at exercising even though that I am not able to walk or run far, but I ride my bicycle. I bike a lot and kind of [pause] walk anyway [laughter] [P3, 12-year-old girl walking] [9].	

		those [persons with SB] that I have met have had a lot more problems than me, with difficulties in walking at all [not audible], so I think I am one of those that are less affected [P3, 12-year-old girl walking] [9].<br><br>Well, I can join them. I can move pretty fast, although not as fast [as the peers] [P1, 10-year-old girl using ambulatory devices/wheelchair] [9]. SB<br><br>one boy's reflection on whether he will be an independent adult: Maybe not completely, but as good as. . .there is always something that you maybe will see as a problem later in life. I do not really know what now, actually [P7, 17-year- old boy in a wheelchair] [9]. SB	

Supplementary File S4: CERQual Summary

No / very minor concerns; [re methodological limitations, coherence, adequacy, relevance] that are unlikely to reduce confidence in review findings.

Minor concerns; [re methodological limitations, coherence, adequacy, relevance] may reduce confidence in review [concerns to be described]

Moderate concerns; [re methodological limitations, coherence, adequacy, relevance] that will probably reduce confidence in review [concerns to be described]

Serious concerns; [re methodological limitations, coherence, adequacy, relevance] that are very likely to reduce confidence in review [concerns to be described]

Summary of review findings	Studies contributing to review finding	Methodological limitations	Coherence	Adequacy	Relevance	CERQUAL assessment of confidence in the evidence	Explanation of CERQUAL assessment
		Concerns about design or conduct of primary studies contributing to review findings.<br>- Design weaknesses<br>- Concerns with data capture<br>- Questions re transparency	Clear and coherent logical fit between data from primary studies and review finding. How well supported or compelling.<br>- How well represented is each review theme<br>- Review theme with weak association or representation.<br>- Do studies provide evidence that looks at area identified.<br>- Audit trail showing reduction in focus?	Determination of degree of richness and quantity of data supporting review finding.<br>- Has review generated theme, subtheme and code?<br>- Has each theme got evidence exploring -ve cases<br>- Is each theme equally saturated with evidence from at least 3 studies.	Extent to which evidence from primary studies is applicable to context in review question.<br>- Evidence of different cultures being used and accounted for.<br>- Similar settings with differences accounted for<br>- Do separate studies consider same PoI and is that represented in study review<br>- Can analysis account for any major differences		
1.1 Challenges to physical activity, sports, or exercise resulting from biological aspects of physical condition.	1, 6, 8, 9	**Minor methodological concerns** [all 4 studies].<br>Unclear influence of researcher on research all studies.	**Minor concerns**<br><br>Well represented by 4 studies out of 9 discuss this theme. Studies	**No Concerns**<br><br>Supported by 4 studies, theme subtheme and code generated.	**Some Concerns**<br><br>All have Spinal Bifida x 4<br><br>Numerous locations	**Minor concerns**	Some lack of information on methodological transparency and influence of researcher on research.

		Unclear congruity between philosophical perspective and research methodology [No. 8 & 9].<br>Unclear location of the researcher culturally and theoretically [No.9].	provide evidence that looks at area identified.	Supported by >3 studies.	- 1 Netherlands<br>- 6 Canada<br>- 8 Canada, rehab hospital<br>- 9 Sweden, OPD		Studies appear well represented and supported.<br><br>All participants have spinal bifida
1.2 Bladder and bowel care and its impact on participation in physical activity.	1, 3, 2, 8, 9	**Minor concerns –** no methodological concerns regarding the design, data collection or capture.	**Minor Concerns**<br>Data appears to be coherent between data in primary studies and review finidngs. Some lack of representation on study 2<br>Fairly well presented across the studies is each review theme. Audit trail visible.	**No Concerns**<br><br>Supported by 5 studies.<br>Equal saturation<br>?one negative case of SCI	**No concerns**<br><br>Evidence of different cultures being used and population of interest represented.	**Very minor concerns**	Some lack of representation on study 2. Data appears well represented with numerous quotes and variety of populations used.
2.1 Feelings of struggle and needing for physical assistance from others. Desire for independence when participating in sports, exercise, and physical activity.	1, 3, 5, 8, 9	**Minor concerns –**<br><br>no methodological concerns regarding the design, data collection or capture. Unclear location of the researcher culturally and theoretically and influence of researcher on research.	**Minor Concerns**<br><br>Studies appear fairly well represented with each review theme visible.	**No concerns**<br><br>Supported by 5 studies, theme subthemes and code generated.	**No concerns**<br><br>Studies relevant to context of review. Evidence from a variety of cultures and population of interest.	**Very minor concerns**	Some concerns regarding influence of researcher on the research. Otherwise no concerns.
2.2 Children and adolescents perceiving themselves as different from their peers, that they do not fit in.	2, 3, 4, 8, 9	**Minor concerns**<br><br>Unclear location of the researcher culturally and theoretically and influence of researcher on research.	**Minor Concerns**<br><br>Some lack of representation on study 2<br>Fairly well presented across the studies is each review theme. Audit trail visible.	**Minor concerns**<br><br>Supported by 5 studies, theme subthemes and code generated.	**Minor Concerns**<br><br>Studies all examine SB with evidence from a variety of cultures and population of interest.	**Minor Concerns**	Concerns regarding coherence and the lack of representation in study 2

2.3 Emotions of anger, fear or sadness towards limited participation in school activities and social events.	2, 3, 6, 7, 8, 9	**Minor concerns**<br><br>Unclear location of the researcher culturally and theoretically and influence of researcher on research.	**Minor Concerns**<br><br>Some lack of representation on study 2 Fairly well presented across the studies is each review theme. Audit trail visible.	**No / Very Minor Concerns**<br><br>Represented by 6 out of 9 studies.	**No / Very Minor Concerns**<br><br>4 studies examine SB, 1 SCI and 1 post op return to PA. All from a variety of cultures and major difference accounted for.	**Very minor concerns**	Concerns regarding coherence and the lack of representation in study 2, and influence of researcher on the research. All other studies well represented and accounted for.
2.4 Need to adjust and accept spinal pain or disability and its influence on participation in sports, exercise, and physical activity.	3, 4, 8, 9	**Minor concerns**<br><br>Unclear location of the researcher culturally and theoretically and influence of researcher on research.	**No / very minor concerns**<br>Subtheme is well represented across the 4 papers with clear coherent fit between primary studies and review findings.	**Minor Concerns**<br><br>No evidence of negative cases	**Minor Concerns**<br><br>All studies examine SB but from a variety of locations and populations within children and adolescents.	**Minor Concerns**	No evidence of negative cases. Data appears otherwise well saturated and adequate with good coherence and relevance.
3.1 Fear and worry towards participation in sports, exercise and PA. Fear towards socialising in a sports group and peer rejection.	2, 4, 7, 8, 9	**Minor concerns**<br><br>Unclear location of the researcher culturally and theoretically and influence of researcher on research.	**Minor Concerns**<br><br>Some lack of representation on study 2 Fairly well presented across the studies is each review theme. Audit trail visible.	**No / Very minor concerns**<br><br>Represented by 5 of 9 studies.	**No / very minor concerns**<br><br>3 studies examining SB, 1 SCI and 1 post op return to PA.	**Minor Concerns**	Concerns regarding coherence and the lack of representation in study 2, and influence of researcher on the research.
3.2 Dependency of child and impact on routine [work, family, time]	1, 3, 4, 9	**Minor concerns**<br><br>Unclear location of the researcher culturally and theoretically and influence of researcher on research.	**No / very minor concerns**<br>Subtheme is well represented across the 4 papers with clear coherent fit between primary studies and review findings.	**No / Very minor concerns**<br><br>Evidence of negative cases and data appears saturated	**Minor Concerns**<br><br>All studies examine SB but from a variety of locations and populations within children and adolescents.	**Minor Concerns**	Evidence of negative cases. All studies examine SB and no other paediatric spinal conditions examined. Subtheme is well represented across the 4 papers.
3.3 Negative attitudes from others and lack of social acceptance. Experiencing ridicule and feeling ashamed.	1, 3, 4, 6, 8, 9	**Minor concerns**<br><br>Unclear location of the researcher culturally and theoretically and	**No / very minor concerns**<br>Clear data from primary studies and review findings	**No / Very minor concerns**<br><br>Data appears saturated and evidence of negative	**Very minor concerns**<br><br>All studies examine SB but across a variety of locations and populations.	**No / very minor concerns**	Some lack of detail of the influence of the researcher on the research. Data is otherwise well represented, all papers

		influence of researcher on research.		cases. Represented by 6 studies.			examine SB but across a variety of locations and populations.
3.4 Friends providing needed emotional support and role models.	1, 3, 4, 5, 6, 8	**Minor concerns**<br><br>Unclear location of the researcher culturally and theoretically and influence of researcher on research.	**No / very minor concerns**<br>Subtheme is well supported and compelling with clear data. Some lack of coherence in study 5 with themes being represented as a percentage rather than a direct quote.	**No / very minor concerns**<br>Data appears saturated and supported by 6 of the 9 studies.	**Minor concerns**<br>5 of the 6 studies examine SB and one achondroplasia.	**Minor concerns**	Some lack of clarity in coherence in study 5 due to indirect participant quotes. Otherwise data is well saturated with good adequacy. Minor concerns regading relevance, one study examining achondroplasia and 5 SB.
3.5 Youth dependent on parents for physical and emotional advice or support.	1, 3, 4, 6, 7, 8, 9	**Minor concerns**<br><br>Unclear location of the researcher culturally and theoretically and influence of researcher on research.	**No / very minor concerns**<br><br>7 out of 9 papers examine this subtheme.	**Minor Concerns**<br><br>No evidence of negative cases but data appears well saturated	**No / very minor concerns**<br><br>5 examine SB, 1 Achondroplasia and 1 post-op return to PA.	**No / Very Minor Concerns**	Data is well saturated with 7 out of 9 papers examining this subtheme. One study examining achondroplasia, another post-operative paediatric spinal surgery and the others spinal bifida.
3.6 Lacking information or support from sports counsellors, local clubs or organisations regarding physical activity.	1, 2, 4, 6	**Minor concerns**<br><br>Unclear location of the researcher culturally and theoretically and influence of researcher on research.	**Minor Concerns**<br><br>Some lack of representation on study 2 Fairly well presented across the studies is each review theme. Audit trail visible.	**Minor Concerns**<br><br>No evidence of negative cases.	**Minor Concerns**<br><br>3 examine SB and 1 SCI.	**Minor Concerns**	Some lack of representation in study 2. No evidence of negative cases and subtheme is represented by 4 of the 9 studies identified.
3.7 Desire to be active and increase environmental access.	1, 4, 8, 9	**Minor concerns**<br><br>Unclear location of the researcher culturally and theoretically and	**No / minor concerns**<br><br>All studies are well represented and subtheme is well supported.	**Minor Concerns**<br><br>No evidence of negative cases but data appears	**No / minor concerns**<br><br>Studies only examine Spinal bifida across a variety of different settings.	**Minor concerns**	Some lack of representation in populations other than spinal bifida affecting the relevance of results. Sub theme is otherwise

		influence of researcher on research.		otherwise well saturated.			well represented and supported by participant quotes with good coherence. No evidence of negative cases influencing adequacy.
